# Fine–mapping of two differentiated thyroid carcinoma susceptibility loci at 2q35 and 8p12 in Europeans, Melanesians and Polynesians

**DOI:** 10.18632/oncotarget.27888

**Published:** 2021-03-02

**Authors:** Julie Guibon, Pierre-Emmanuel Sugier, Om Kulkarni, Mojgan Karimi, Delphine Bacq-Daian, Céline Besse, Anne Boland, Elisabeth Adjadj, Frédérique Rachédi, Carole Rubino, Constance Xhaard, Claire Mulot, Pierre Laurent-Puig, Anne-Valérie Guizard, Claire Schvartz, Rosa Maria Ortiz, Yan Ren, Evgenia Ostroumova, Jean-François Deleuze, Marie-Christine Boutron-Ruault, Ausrele Kesminiene, Florent De Vathaire, Pascal Guénel, Fabienne Lesueur, Thérèse Truong

**Affiliations:** ^1^University Paris-Saclay, UVSQ, Inserm, Gustave Roussy, CESP, Exposome and Heredity Team, Villejuif, France; ^2^Inserm, U900, Institut Curie, PSL University, Mines ParisTech, Paris, France; ^3^University Paris-Saclay, CEA, Centre National de Recherche en Génomique Humaine, Evry, France; ^4^University Paris-Saclay, UVSQ, Inserm, Gustave Roussy, CESP, Epidemiology of Radiations Team, Villejuif, France; ^5^Endocrinology Unit, Territorial Hospital Taaone, Papeete, French Polynesia; ^6^University of Lorraine, INSERM CIC 1433, Nancy CHRU, Inserm U1116, FCRIN, INI-CRCT, Nancy, France; ^7^Centre de Recherche des Cordeliers, INSERM, Sorbonne Université, USPC, Université Paris Descartes, Université Paris Diderot, EPIGENETEC, Paris, France; ^8^Registre Général des Tumeurs du Calvados, Centre François Baclesse, Caen, France; ^9^Inserm U1086 -UCN "ANTICIPE", Caen, France; ^10^Registre des Cancers Thyroïdiens, Institut Godinot, Reims, France; ^11^Institute of Oncology and Radiobiology, La Havana, Cuba; ^12^International Agency for Research on Cancer, Lyon, France; ^*^These authors contributed equally to this work

**Keywords:** thyroid cancer, cancer genetics, case-control study, fine-mapping study, single nucleotide polymorphism

## Abstract

Differentiated thyroid carcinoma (DTC) incidence is characterized by wide ethnic and geographic variations, with high incidence rates observed in Oceanian populations. Genome-wide association studies (GWAS) identified mainly four DTC susceptibility loci at 9q22.33, 14q13.3, 2q35 and 8p12. Here we performed fine-mapping of the 2q35 and 8p12 loci in the population of the EPITHYR consortium that includes Europeans, Melanesians and Polynesians to identify likely causal variants for DTC risk. We conducted a colocalization analysis using eQTLs data to determine the SNPs with the highest probability of causality.

At 2q35, we highlighted rs16857609 located in *DIRC3*. This SNP has a high probability of causality in the three populations, and a significant association in Europeans (OR = 1.4, *p* = 1.9 x 10^-10^). It is also associated with expression of *DIRC3* and of the nearby gene *IGFBP5* in thyroid tumour cells. At 8p12, we identified rs7844425 which was significantly associated with DTC in Europeans (OR = 1.32, *p* = 7.6 x 10^-8^) and rs2439304, which was highlighted by the colocalization analysis but only moderately associated with DTC in our dataset (OR = 1.2, *p* = 0.001). These SNPs are linked to the expression of *NRG1* in thyroid tissue.

Hence, our study identified novel variants at 2q35 and 8p12 to be prioritized for further functional studies.

## INTRODUCTION

Thyroid cancer (TC) is the most common endocrine malignancy. Its incidence has been rising over the past few decades and varies considerably around the world. Elevated incidence was reported in some Pacific islands such as French Polynesia and New-Caledonia, two French territories, with estimated incidence rates in 2018 being 27.7/100,000 and 25.6/100,000 person-years (PY) in women and 6.5/100 000 and 7.6/100 000 PY in men [1], respectively. Ethnic differences in incidence have also been reported in New-Caledonia with higher rates among Melanesians than in other ethnic groups [2]. In metropolitan France, the incidence rates were 20.9/100,000 in women and 5.7/100,000 PY in men in 2018 [1].

Papillary thyroid carcinoma (PTC) and follicular thyroid carcinoma (FTC) are the most frequent subtypes of differentiated thyroid carcinoma (DTC), and represent about 90% of all TC. Apart from exposure to ionizing radiation during childhood which is the only well-established risk factor, DTC risk has been consistently associated with obesity [3]. Other risk factors such as deficiency or excess of iodine levels [4] or some hormonal and reproductive factors are also suspected [5]. Moreover, familial risk for TC is among the highest of all cancers [6], suggesting a role of genetic risk factors. Six previous genome-wide association studies (GWAS) of DTC conducted in European populations [7–12] and a GWAS conducted in the Asian population [13] allowed the identification of several DTC susceptibility loci, with the strongest associations being reported at 9q22.33, 14q13.3, 2q35 and 8p12. Recently, a meta-analysis including 3,001 DTC cases and 287,500 controls of European descent confirmed these findings and yielded five additional loci associated to DTC at 1q42.2, 3q26.2, 5q22.1, 10q24.33, 15q22.33 that were not replicated yet [12]. We recently conducted a GWAS in EPITHYR that confirmed the susceptibility loci at 9q22.33, 14q13.3, 2q35 and 8p12, and suggested novel signals at 1p31.3 and 16q23.2 [14].

Fine-mapping studies have already been conducted to identify potential causal variants at loci 9q22.33 and 14q13.3 [15, 16]. These variants were subsequently widely replicated, and functional studies were performed to support their role in the aetiology of DTC. To our knowledge, no fine-mapping analyses have been performed so far at 2q35, 8p12 or other DTC susceptibility loci.

Rs966423 at 2q35 and rs2439302 at 8p12 were first identified in GWAS to investigate genetic factors associated with thyroid stimulating hormone (TSH) levels. In the study conducted in Iceland on 27,758 individuals with available circulating TSH measurements [9], the identified SNPs were also found to be associated with DTC risk [9]. Subsequently, these variants were replicated in other populations of European [9, 12] and Asian [13] ancestry.

At locus 2q35, several other variants were also highlighted by GWAS to be associated with DTC in populations of European ancestry. Rs6759952 was reported in GWAS conducted on 3648 DTC cases and 4,224 controls from Italy [10], while a recent meta-analysis of GWAS reported the strongest association with variant rs11693806 at this locus [12]. In European populations, both rs11693806 and rs6759952 were moderately correlated with rs966423 (with r² = 0.47 and r² = 0.74, respectively) [10, 12]. In Korea, a GWAS identified two additional SNPs at 2q35, namely rs12990503 and rs1549738, associated with DTC risk [13]. According to 1000Genomes, rs12990503 was highly correlated (r² = 0.97) with rs11693806 in East Asian populations while rs1549738 was moderately correlated with the GWAS SNP rs966423 (r² = 0.41). Most of the risk variants at this locus are located within the *DIRC3* (*disrupted renal cancer 3*) gene. Although this gene is presumed to have a tumour suppressor activity [10], no functional studies have been performed so far to validate this hypothesis. In 2012, Gudmundsson *et al.* hypothesized that *DIRC3* variants by reducing TSH levels, may lead to reduced differentiation of the thyroid epithelium, and consequent predisposition to malignant transformation [9].

At locus 8p12, only rs2439302 and rs2466076 have been associated with DTC risk in European populations, and the two SNPs were reported as strongly correlated (r² = 0.94 in Icelandic population) [12]. The Korean GWAS highlighted two other DTC risk variants, namely rs6996585 and rs12542743 [13], with rs6996585 being moderately correlated with the GWAS SNP rs2439302 (r^2^ = 0.67 in Asian population of 1000Genomes). All associated variants at this locus were located within the *NRG1* gene, which encodes neuregulin 1, a signalling protein that mediates cell-cell interactions and plays a role in the development of multiple organ systems.

Altogether, several DTC risk variants have been identified at 2q35 and 8p12 but few of them have been further explored with fine-mapping or functional studies to identify the causal ones. Here, our goal was to perform a fine-mapping analysis of these two loci using genotypic data from the EPITHYR GWAS involving Europeans, Polynesians and Melanesians, three populations with distinguishable genetic architecture, and functional annotations to highlight the potential causal variants.

## RESULTS

We used data from EPITHYR consortium in which GWAS data was available for 1,855 cases and 2,321 controls of Europeans, Polynesians and Melanesians ancestries. The distribution of the study participants by study, age groups, sex and TC histology is presented in Table 1 for each ethnic group. Women represented more than 82% of cases in each ethnic group. As expected, the majority of cases were PTC (> 82% of cases in each ethnic group). The number of SNPs included in the current analysis at loci 2q35 and 8p12 are provided for each ethnic group in Supplementary Table 1.

**Table 1:** Characteristics of subjects from EPITHYR included in the analyses by ethnic group

	Europeans				Polynesians				Melanesians			
	Cases		Controls		Cases		Controls		Cases		Controls	
	*N* (1554)	**%**	*N* (1973)	**%**	*N* (146)	**%**	*N* (242)	**%**	*N* (155)	**%**	*N* (106)	**%**
**Study**												
**CATHY**	451	*28.9*	534	*27.1*	1	*0.7*						
**Cuba**	102	*6.6*	113	*5.7*								
**Chernobyl**	66	*4.2*	304	*15.4*								
**E3N**	277	*17.8*	287	*14.5*								
**New Caledonia**	21	*1.3*	70	*3.5*	28	*19.2*	55	*22.7*	155	*100*	106	*100*
**French Polynesia**			4	*0.2*	114	*78.1*	181	*74.8*				
**YOUNG-thyr**	637	*41.0*	661	*33.5*	3	*2.0*	6	*2.5*				
**Age (years)**												
**< 25**	272	*17.5*	513	*26.0*	0	*0*	0	*0*	0	*0*	0	*0*
**25–50**	763	*49.1*	887	*45.2*	113	*77.4*	186	*73.3*	89	*57.4*	69	*65.1*
**≥ 50**	519	*33.4*	573	*29.0*	33	*22.6*	56	*26.7*	66	*42.6*	37	*34.9*
**Sex**												
**Men**	275	*17.7*	454	*23.0*	14	*9.6*	22	*8.6*	9	*5.8*	8	*7.5*
**Women**	1279	*82.3*	1519	*77.0*	132	*92.4*	220	*91.4*	146	*94.2*	98	*92.5*
**Histology**												
**Papillary**	1417	*91.2*			121	*82.9*			132	*85.2*		
**Follicular**	137	*8.8*			25	*17.1*			23	*14.8*		
**Size of carcinoma (only papillary)**												
**< 10 mm**	506	*32.6*			67	*45.9*			69	*44.5*		
**≥ 10 mm**	904	*58.2*			45	*30.8*			62	*40.0*		
**Missing**	144	*9.2*			34	*23.3*			24	*15.5*		

### Locus 2q35

#### Fine-mapping

**Figure 1:**
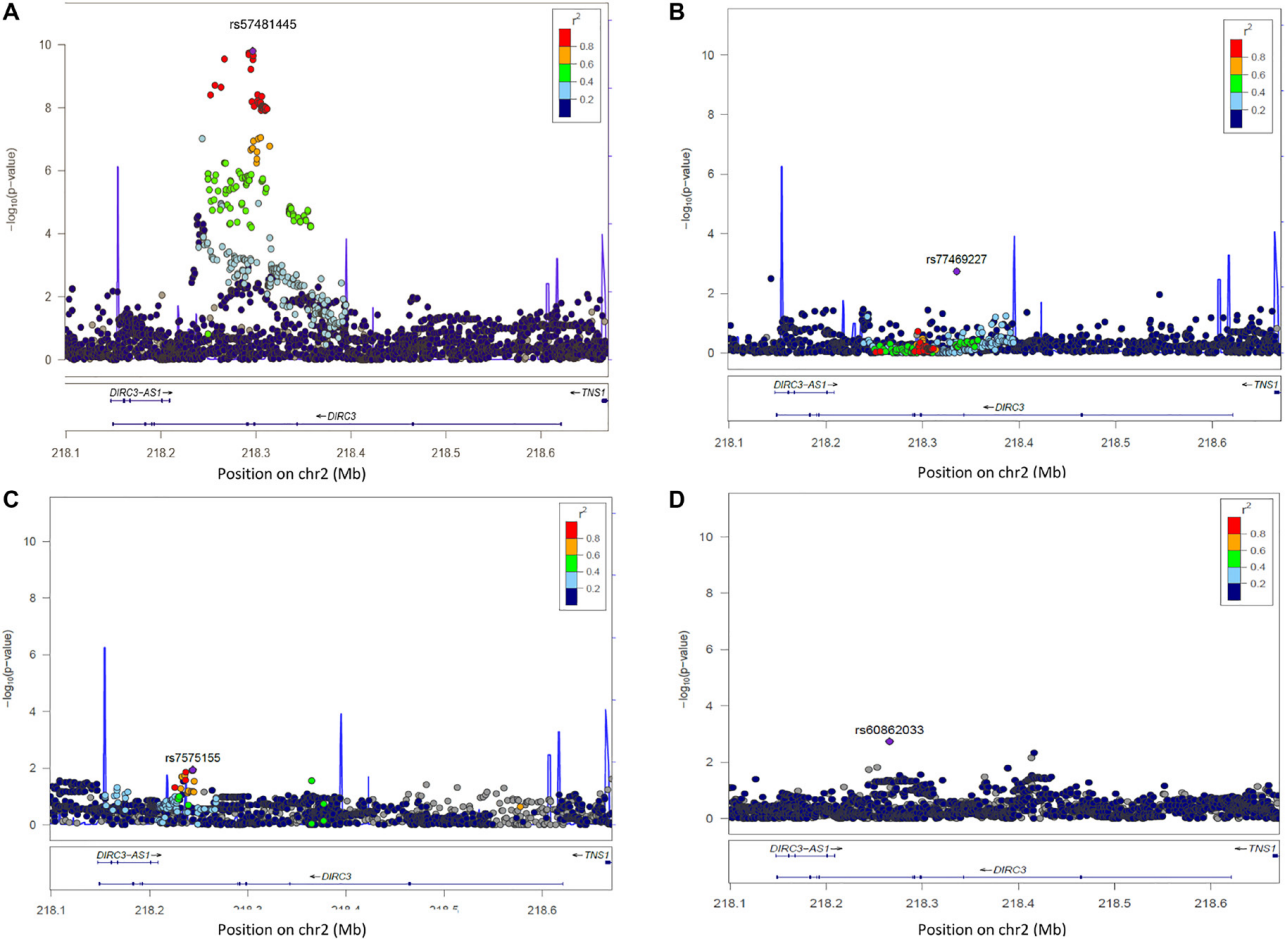
Regional plot of single-SNP association and conditional regression at locus 2q35 in Europeans, Melanesians and Polynesians. On Y axis, are the –log10 (*p*-value); on X axis, the position of SNPs and genes on the chromosome. The color of each SNP spot reflects its r² with the most associated SNP (in purple). The correlation between SNP is ranged from high (in red) to low (in blue). Grey dots correspond to missing variants (imputed SNPs with no genotype with probability > 0.9). (**A**) Plot of –log10 (*p*-values) of SNP association results at locus 2q35 in Europeans. (**B**) Plot of –log10 (*p*-values) of SNP association results at locus 2q35 in Europeans, after conditioning the test on SNP rs57481445. (**C**) Plot of –log10 (*p*-values) of SNP association results at locus 2q35 in Melanesians. (**D**) Plot of –log10 *p*-values of SNP association results at locus 2q35 in Polynesians.

In Europeans, among the 2,682 analysed SNPs at 2q35, 42 SNPs were associated with DTC risk at *p* < 5 × 10^-8^ (Supplementary Table 2). We replicated the associations from previous GWAS with SNPs rs6579952 (OR = 1.26, *p* = 2.9 × 10^-10^), rs11693806 (OR = 1.43, *p* = 1.1 × 10^-10^), rs1382435 (OR = 1.32, *p* = 1.2 × 10^-7^) and rs966423 (OR = 1.27, *p* = 2.6 × 10^-6^) (Supplementary Table 3). However, in our sample, the most significant association was observed for the genotyped SNP rs57481445 (OR = 1.43, *p* = 8.6 × 10^-11^) (Figure 1A and Supplementary Table 2). In Europeans, this variant was strongly correlated with rs11693806 (r^2^ = 0.98) and 38 of the most significantly associated SNPs (Supplementary Table 4). No significant additional signal was observed after conditioning the association test on the top SNP rs57481445 (Figure 1B), suggesting that any residual association signal due to some additional independent SNPs was unlikely within the region.

In Melanesians and Polynesians, no SNP reached the standard genome-wide significance *P*-value threshold of 5 × 10^-8^ (Figure 1C and 1D) and the four previously reported associated SNPs in European GWAS, rs6759952, rs1169s3806, rs1382435 and rs966423, were not replicated here (Supplementary Table 3). Unlike the European population, very high effect allele frequencies were observed for these SNPs. The most significant SNPs in this locus were rs7575155 in Melanesians (OR = 2.42, *p* = 1.1 × 10^-2^) and rs60862033 (OR = 6.3, *p* = 1.8 × 10^-3^) in Polynesians (Figure 1C and 1D).

The LD patterns at 2q35 in the three ethnic groups were slightly similar with LD blocks of stronger LD between SNPs in Melanesians and Polynesians (Supplementary Figure 1). The identified SNPs rs57481445, rs16857609 and rs3821098 were located within the same LD block in the three populations.

In the pooled analysis, the most significant association at locus 2q35 was observed for the genotyped SNP rs3821098 (OR = 1.44, *p* = 1.6 × 10^-12^) (Supplementary Figure 2A) which is in strongly correlated to rs57481445 (r^2^ > 0.87 in the three populations). After conditioning on the top SNP rs3821098, no additional SNP was highlighted (Supplementary Figure 2B). Of note, although only imputed SNPs with an imputation quality score above 0.3 were kept in the analyses, all highlighted variants had info scores above 0.9.

### Functional data

The colocalization analysis using eQTL data (Supplementary Figure 3A) highlighted two intronic *DIRC3* variants, namely rs16857609 (PP of causality = 0.41) and rs12990503 (PP of causality = 0.39) (Table 2 and Supplementary Figure 3B). These two SNPs were highly correlated to rs57481445 (r^2^ > 0.9) and also strongly associated with the expression of *DIRC3* and *IGFBP5* (insulin growth factor binding protein 5) in thyroid tumour cells, according to TCGA data [17].

**Table 2:** Results of colocalization analyses for SNPs with posterior probability of causality > 0.10 at loci 2q35 and 8p12, in Europeans, Polynesians, Melanesians and all individuals

Locus	SNP	EA	Europeans				Polynesians				Melanesians				eQTL	
			EAF	OR	*p*	PP	EAF	OR	*p*	PP	EAF	OR	*p*	PP	P^(b)^	associated gene
2q35	rs16857609	T	0.28	1.42	3.7 × 10^-10^	0.41	0.82	1.42	0.12	0.32	0.92	1.05	0.88	0.22	1.9 × 10^-20^ 2.8 × 10^-19^	*DIRC3* *IGFBP5*
	rs12990503	C	0.28	1.41	3.4 × 10^-10^	0.39	0.17	1.48	0.35	0.35	0.08	0.90	0.75	0.39	1.9 × 10^-20^ 1.2 × 10^-19^	*DIRC3* *IGFBP5*
8p12	rs2439304^(a)^	A	0.50	1.16	1.7 × 10^-3^	0.65	1.11	1.11	0.52	0.52	0.41	1.03	0.84	0.35	3.1 × 10^-39^	*NRG1*

EA: effect allele; EAF: effect allele frequency; OR: odds ratio; p: *p*-value; PP: posterior probability of causality. PP were derived from the colocalization analysis (using eQTL *p*-values obtained from PancanQTL database). (a) Imputed SNP, the quality score of imputation was equal to 1 for the 3 ethnic groups. (b) *P*-values of eQTLs of thyroid tumor cells from PancanQTL database.

In Melanesians and Polynesians, the colocalization analysis also gave the highest PP of causality values for the same two SNPs identified in Europeans: rs16857609 with a PP_Melanesians_ = 0.22 and PP_Polynesians_ = 0.32, and rs12990503 with a PP_Melanesians_ = 0.39 and PP_Polynesians_ = 0.35 (Supplementary Figure 3C and 3D). Rs16857609 and rs12990503 were strongly correlated in the three ethnic groups with r² > 0.88.

Rs57481445 and rs16857609, very close to each other (< 240 bases) and located within the same LD block in the three populations (Supplementary Figure 1), are mostly located within the same functional annotation sites. They are both located within sites of histone modification, notably sites of epigenetic acetylation and methylation on histone H3 protein subunit. They are also located within markers of enhancer chromatin state and actively transcribed chromatin states, which are active states associated with genes’ expression level in adipose tissue and adipocytes. Furthermore, rs16857609 is located within sites of DNase involving in three different cellular types (ovary, fetal heart and pluripotent cells). Rs12990503 is located within markers of chromatin state in adipocytes which are low or weak transcription site of gene.

### Stratified analyses

We performed subgroup analyses on rs16857609 in strong LD with the top SNP rs57481445 in the three ethnic groups because it was the most interesting candidate SNP with regards to its functional annotation data. Stratified analyses were performed by study, sex, age group, TC histology and size of papillary carcinoma (Figure 3A). Heterogeneity was observed when stratifying by tumour histology (with I² = 99.2%, *p* < 10^-4^). We found that carriers of the risk allele [T] have a higher risk of developing PTC rather than FTC. No heterogeneity was detected in Melanesians and Polynesians. Although association was non-significant in these two populations, ORs associated with this allele were in the same direction in the three populations. Interestingly, we observed that the risk allele [T] was more common in Oceanians (allele frequency = 0.92 and 0.82 in Melanesians and Polynesians, respectively) than in Europeans (allele frequency = 0.28).

**Figure 2:**
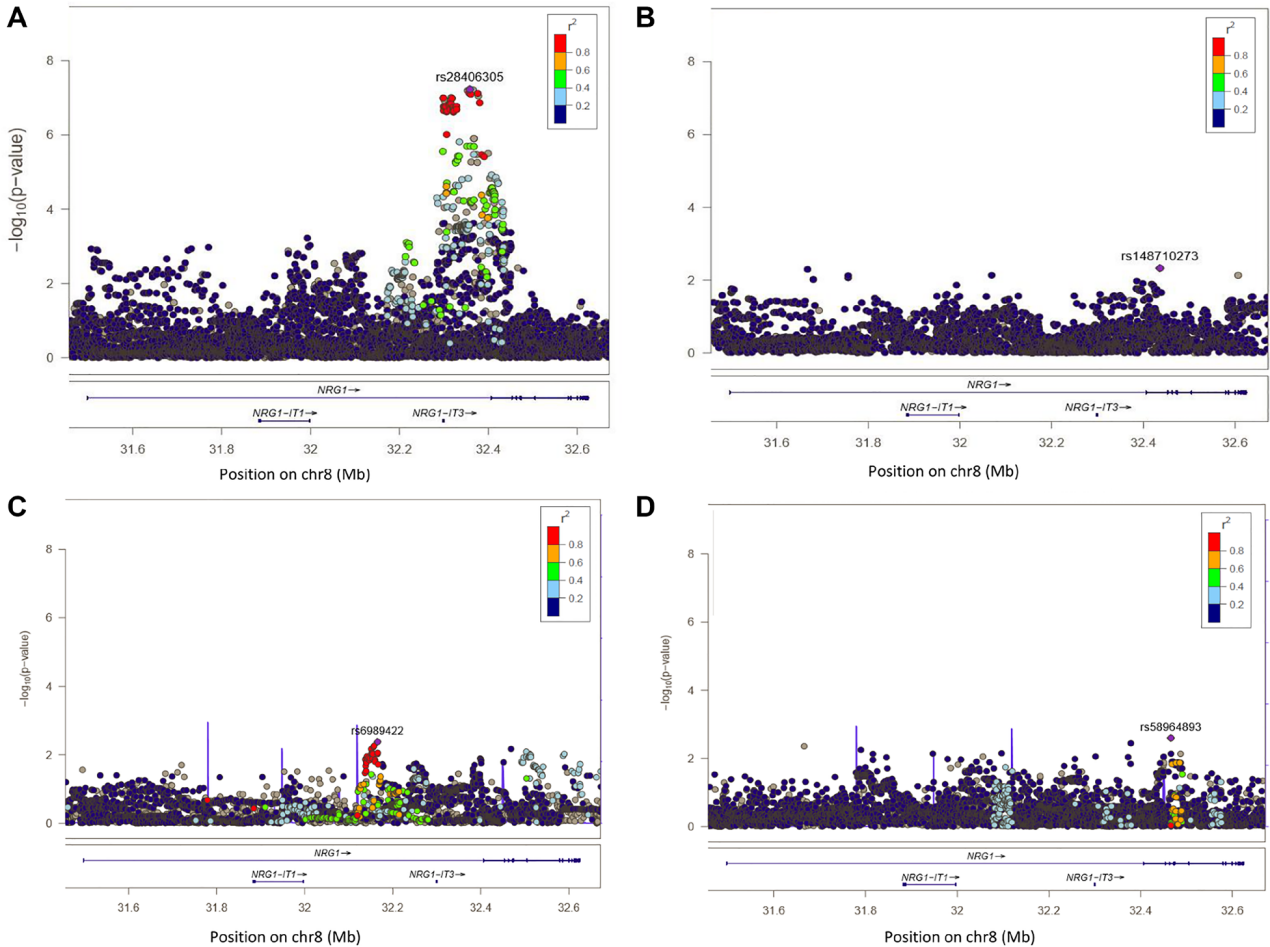
Regional plot of single-SNP association and conditional regression at locus 8p12 in Europeans, Melanesians and Polynesians. On Y axis, are the –log10 (*p*-value); on X axis, the position of SNPs and genes on the chromosome. The color of each SNP spot reflects its r² with the most associated SNP (in purple). The correlation between SNP is ranged from high (in red) to low (in blue). Grey dots correspond to missing variants (imputed SNPs with no genotype with probability > 0.9). (**A**) Plot of –log10 (*p*-values) of SNP association results at locus 8p12 in Europeans. (**B**) Plot of –log10 (*p*-values) of SNP association result rs28406305 at locus 8p12 in Europeans, after conditioning the test on SNP rs28406305. (**C**) Plot of –log10 (*p*-values) of SNP association results at locus 8p12 in Melanesians. (**D**) Plot of –log10 (*p*-values) of SNP association results at locus 8p12 in Polynesians.

**Figure 3:**
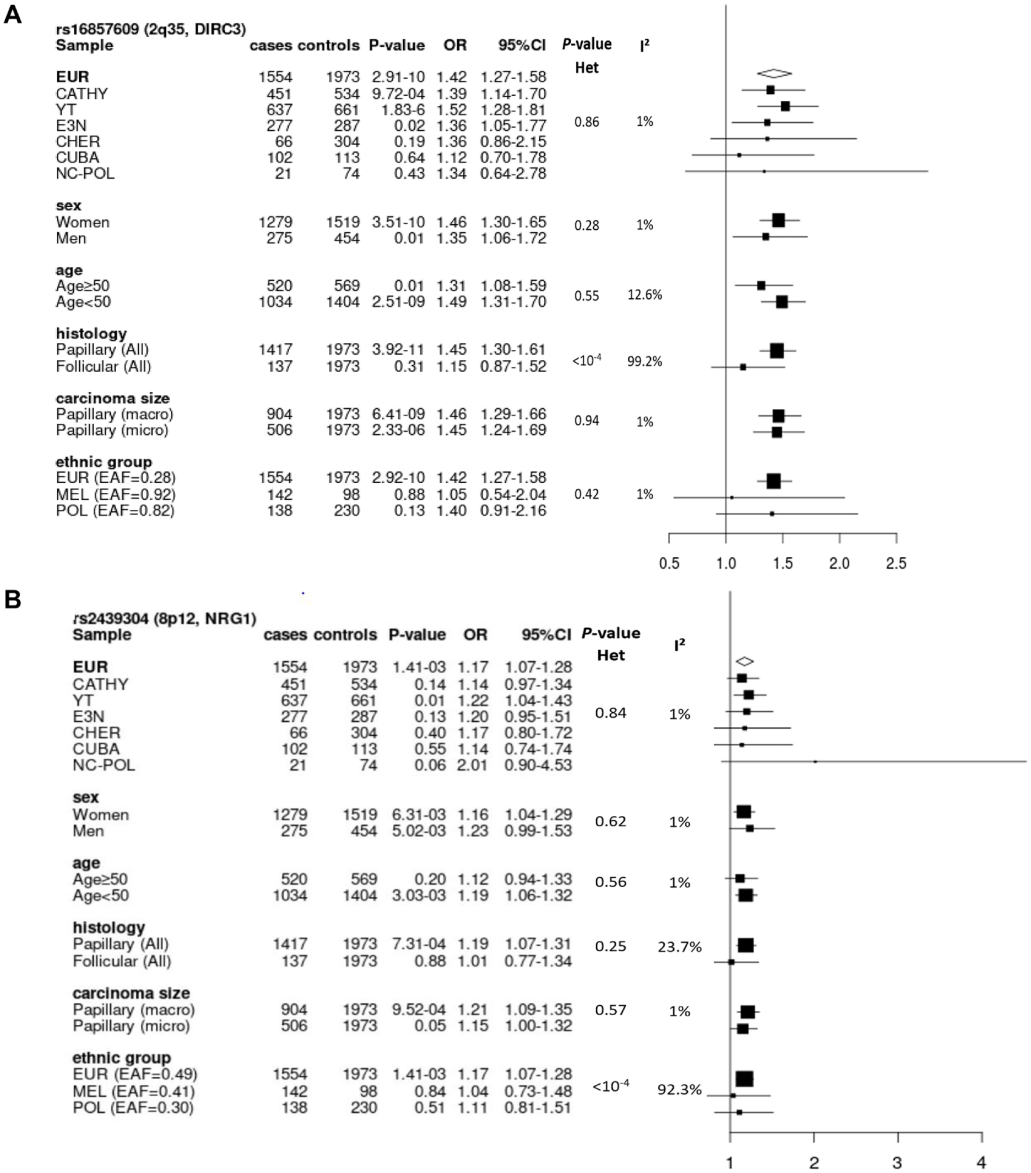
Forest plots of subgroup analyses for rs16857609 (**A**) and rs2439304 (**B**), by study, sex, age group, histology and size of carcinoma in Europeans, and by ethnic group in the entire dataset. (A) Result for the genotyped SNP rs16857609 in *DIRC3* at 2q35 (Effect allele (T) frequency in Europeans: 0.28). (B) Result for the imputed SNP rs2439304 in *NRG1* at 8p12 (Effect allele (A) frequency in Europeans: 0.49). EAF: effect allele frequency; EUR: Europeans; MEL: Melanesians; POL: Polynesians; Het: heterogeneity.

### Locus 8p12

#### Fine-mapping

At locus 8p12, we replicated the previously reported associations with SNPs rs2466076 (OR = 1.21, *p* = 1.0 × 10^-4^) and rs2439302 (OR = 1.20, *p* = 2.6 × 10^-4^) in Europeans. Furthermore, rs6996585 and rs1254274, identified in previous GWAS in Asians, were also associated with DTC risk in Europeans from EPITHYR (OR = 1.21, *p* = 1.6 × 10^-4^, and OR = 1.22, *p* = 9.8 × 10^-5^ respectively) (Supplementary Table 3). However, none of these associations reached a genome-wide significance level (*p* < 5 × 10^-8^). In Melanesians and in Polynesians, none of these SNPs were significant but the OR estimates for these four previously reported SNPs were in the same direction than for the Europeans (Supplementary Table 3).

In Europeans, the strongest association was observed for the genotyped SNP rs28406305 (OR = 1.32, *p* = 5.9 × 10^-8^) and 37 SNPs were associated with DTC risk at *p* < 1 × 10^-7^ (Supplementary Table 5). In our European sample, this variant was moderately correlated with the SNP rs6996585 highlighted in a previous GWAS [13] with pairwise r² = 0.35. After conditioning the association test on the lead SNP rs28406305 (Figure 2A), no additional significant signal was observed (Figure 2B) suggesting the presence of only one causal variant within the region.

In Melanesians and Polynesians, no SNP was significantly associated with DTC (Figure 2C and 2D). The most significant association was observed for rs6989422 in Melanesians (OR = 2.06, *p* = 4.1 × 10^-3^) and for rs58964893 (OR = 1.71, *p* = 2.5 × 10^-3^) in Polynesians. In these populations, these two SNPs were not correlated with rs28406305 which was highlighted in Europeans (r² < 0.01).

The LD patterns at 8p12 in the three ethnic groups were similar, with larger LD blocks observed in Melanesians (Supplementary Figure 4). However, the SNPs rs2439304 and rs28406305 were located in the same LD block in Melanesians but not in Europeans nor in Polynesians.

In the pooled analysis, the most significant association was observed for the genotyped SNP rs28406305 (OR = 1.30, *p* = 4.1 × 10^-8^). After conditioning the analysis on the top SNP rs28406305, no additional SNP was highlighted (Supplementary Figures 2C and 2D).

### Functional data

Using eQTL data from PancanQTL, the colocalization analysis gave the highest PP for rs2439304 in the three ethnic groups (PP_Europeans_ = 0.65, PP_Melanesians_ = 0.52 and PP_Polynesians_ = 0.35) (Table 2 and Supplementary Figure 5). In these three groups, this SNP was strongly correlated with the GWAS SNP rs2439302 (r² = 1), but moderately correlated with the top SNPs (r^2^ = 0.50 with rs28406305 in Europeans, r^2^ = 0.40 with rs6989422 in Melanesians and r^2^ = 0.59 with rs58964893 in Polynesians). Furthermore, this SNP was not significantly related to DTC (in Europeans: OR = 1.20, *p* = 2.6 × 10^-4^; in Polynesians: OR = 1.10, *p* = 0.55; in Melanesians: OR = 1.05, *p* = 0.79). Also, rs2439304 is an eQTL strongly associated with the expression of *NRG1* in thyroid tumor cells with *p* = 3.1 × 10^-39^ in PancanQTL. Rs2439304 is located in the promoter region of *NRG1*. It alters many enhancer sites of histone modifications and markers of chromatin states in several tissues. This SNP is notably located within two markers of actively transcribed chromatin states, which are active states associated with genes’ expression level in adipocytes. Rs2439304 is also located within enhancer site of epigenetic methylation to the DNA packaging protein Histone H3 in adipocytes.

Rs28406305, which is the top SNP in Europeans, is located within two sites of epigenetic methylation to the DNA packaging protein Histone H3 H3K4me3 in adipocytes and liver cells. Among the top SNPs that are in the same LD block than rs28406305, rs7844425 (r^2^ = 0.96, OR = 1.32, *p* = 7.6 × 10^-8^) was more likely to have a deleterious impact based on the probability score of being likely a regulatory variant calculated with RegulomeDB and the results of Haploreg V4. This SNP is located within seven sites of epigenetic methylation to H3K4me3 in several tissues, within two sites of epigenetic acetylation to the DNA packaging Histone H3 H3K27ac, and within several markers of chromatin states in different tissues. Importantly, rs7844425 is an eQTL strongly associated with the expression of *NRG1* in thyroid tissue, with *p* = 2.3 × 10^-17^ in PancanQTL. However, this SNP was not highlighted in the colocalization analysis, with very low PP in the three ethnic groups (PP_Europeans_ = 8.8 × 10^-28^, PP_Melanesians_ = 3.4 × 10^-27^ and PP_Polynesians_ = 3.4 × 10^-27^).

### Stratified analyses

We performed subgroup analyses for rs2439304, which had the highest PP of causality based on eQTL data, by stratifying by study, sex, age group, histology and size of the thyroid carcinoma in Europeans (Figure 3B). No heterogeneity by subgroup was observed. However, while comparing the results by ethnic groups, we observed a significant heterogeneity between these groups (I² = 92.3%, *p* < 10^-4^). This variant was imputed with high imputation quality in all populations (info score > 0.99).

**Figure 4:**
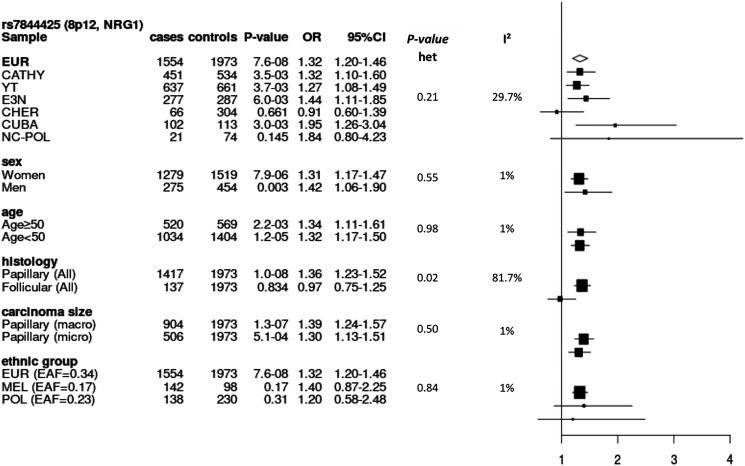
Forest plot of subgroup analyses for rs7844425 stratifying on study, sex, age group, histology and size of carcinoma in Europeans, and stratifying on population group in the entire dataset. Result for the imputed SNP rs7844425 in *NRG1* at 8p12 (Effect allele (A) frequency in Europeans: 0.34). EAF: effect allele frequency; EUR: Europeans; MEL: Melanesians; POL: Polynesians; Het: heterogeneity.

We also performed subgroups analyses for rs7844425 which was the most functionally interesting SNP associated to DTC based on the results of Regulome DB and Haploreg V4. In Europeans we observed a significant heterogeneity by histological type of DTC (I² > 81%, *p* = 0.02), with a positive association observed only for PTC (Figure 4).

## DISCUSSION

The few GWAS conducted so far allowed the identification of several DTC susceptibility loci with the strongest associations located on 2q35, 9q22.33, 8p12 and 14q13.3. Published fine-mapping studies have been conducted at loci 9q22.22 and 14q13.3 [15, 16], including our previous study investigating loci 9q22.33 and 14q.13.3 in Melanesians [15], but the present study is, to our knowledge, the first study that investigates loci 2q35 and 8p12 in details. Moreover, investigating these genomic regions not only in Europeans but also in Melanesians and Polynesians from New-Caledonia and French Polynesia is of particular relevance given that these populations have one of the highest incidence rates of DTC in the world. Although high parity and obesity have been associated with DTC risk in Melanesians [18, 19] and could explain a part of the elevated incidence, the remaining reasons are yet unknown. Therefore, deciphering the genetic architecture of DTC in these under-studied populations could shed new light on the susceptibility to this endocrine-related cancer.

The trans-ethnic aspect is an asset of the EPITHYR study. Indeed, it was showed that trans-ethnic meta-analyses that combine results of the same trait across genetically diverse populations can aid fine-mapping by capitalizing on ethnic differences in LD patterns [20]. The LD patterns at 2q35 and 8p12 in the three ethnic groups were slightly similar although LD blocks were larger in Melanesians and Polynesians than in Europeans (Supplementary Figures 1 and 4). However, the limited sample size of the Polynesian and Melanesian populations led to a lack of power to detect associations. In order to gain statistical power, we chose to conduct a pooled analysis using LMM, so that the differences in LD patterns between the individuals were taken into account by adjusting for the genetic relationship matrix as random effect.

For the two investigated loci, association between DTC risk and the previously reported GWAS SNPs were replicated in Europeans (Supplementary Table 3). The PCA performed in Polynesians and in Melanesians using the HGDP reference panel showed that Polynesians are genetically close to East Asians (Supplementary Figure 6). Therefore, despite the lack of power, we could have expected to replicate the associations previously highlighted in Asians, among Polynesians from EPITHYR and to a lesser extend in Melanesians. At locus 8p12, although the results were not statistically significant, the reported ORs in these two populations were in the same direction than those previously reported in the Asian GWAS. However, at locus 2q35, the previously associated SNPs in Asian GWAS were in the same direction in Polynesians but not in Melanesians (Supplementary Table 3).

In Europeans, the conditional analysis showed that the association of DTC risk with the top SNP rs57481445 was correlated with other SNPs within the region, suggesting that only one independent signal lies at 2q35. Our analyses pinpointed rs16857609 as a possible causal SNP for this region in Europeans, Melanesians and Polynesians. This SNP was strongly associated with the expression of the two nearby genes *DIRC3* and *IGFBP5* in thyroid tumour cells, according to TCGA data. Insulin-like growth factors (IGFs) play a pivotal role in tissue homeostasis, regulating cell proliferation, differentiation and migration during development and also in adulthood. Since it was shown that *IGFBP5* is overexpressed in PTC, it could have a proliferative effect on thyroid tumour cells [21]. We found that rs16857609 is located within different methylation sites involving histone H3 in many tissues. Also, this SNP is located within many regulatory elements (chromatin state linked to an enhancer, histone modifications in DNA methylation or acetylation) that control genes expression in adipose nuclei tissue and in adipocyte stem cells. Since obesity has been consistently associated with an increased risk of TC in epidemiological studies [3], these observations support the hypothesis that rs16857609 could play a role in the aetiology of DTC via complex obesity-associated pathways. The frequency of the risk allele [T] is much higher in Oceanians than in Europeans and this could explain a part of the highest incidence of DTC observed in the former populations if the association is confirmed. Interestingly, rs16857609 has been associated with increased breast cancer risk in Europeans [22]. Since IGFBP5 is a physiological regulator of epithelial cell survival in the mammary gland [23], it was proposed that a deregulation of *IGFBP5* expression induced by variants located upstream of *DIRC3* [24] could lead to the development of a breast tumour. Altogether, the link between rs16857609, breast cancer risk and IGFBP5 on one side, and the link between TC and IGFBP5 on the other suggests that a deregulation of IGFBP5 expression may also lead to the proliferation of thyroid cells and eventually to PTC development.

The conditional analysis at locus 8p12 in Europeans revealed a single association signal. Among the most associated SNPs, we reported rs7844425 as the variant which is the most likely to have a functional deleterious impact. This SNP is located in the upstream transcript region of the major isoforms of the *NRG1* gene expressed in human thyroid tissue and thyroid cell lines, namely NM_004495, NM_013958 and NM_001160008. The gene encodes a membrane glycoprotein that mediates cell-cell signalling and plays a critical role in the growth and development of multiple organ systems. Rs7844425 is predicted to alter conserved elements involved in the regulation of *NRG1* expression. To our knowledge, no previous GWAs highlighted rs7844425 or other SNPs from our study located in the same LD block. Interestingly, this SNP is strongly correlated with rs17716295 in Europeans (r² = 0.93) which was associated with antipsychotic treatment response in schizophrenia patients [25] and it was recognized that disturbances in the balance of thyroid hormones contribute to mental status deregulation recognized that thyroid dysfunction is associated with psychiatric disorders, with a higher proportion of hypothyroidism among patients with schizophrenia [26]. We found that rs7844425 is only moderately correlated with rs2439304 that we also highlighted at the locus 8p12 (r^2^ = 0.51 in European). Although rs2439304 was not significantly associated with DTC in our populations (OR = 1.2, *p* = 0.001 in Europeans), it had the highest PP of causality in the three ethnic groups using eQTL data at 8p12. Rs2439304 is strongly correlated with rs2439302 which was reported in a previous GWAS on DTC risk [27] (r^2^ = 1). Both SNPs are located within a 32kb LD block in intron 1 of *NRG1*. By performing haplotype analysis of this LD block, He *et al.* proposed that the impact of several risk alleles on transcriptional regulation is probably combinatorial in dictating *NRG1* expression and in conferring susceptibility to DTC. Furthermore, by using CHIP-seq experiment and luciferase assay in primary thyroid cell culture, they showed that the risk allele of rs2439304 and the risk allele of rs2439302 increase expression of all three major NRG1 isoforms when comparing with the wild type allele. This result is in line with the eQTL analysis conducted by Son *et al.* suggesting that the increased expression of *NRG1* in thyroid tissue might influence the development or progression of TC [13] but do not support results by Gudmundsson *et al.* who reported that carriers of rs2439302 risk allele have a lower expression of *NRG1* [9].

For both SNPs, rs7844425 and rs16857609, we performed analyses restricted to PTC as sensitivity tests but results did not differed significantly from the overall analysis so we chose not to present these results (data not shown).

In summary, we confirmed the previously associated SNPs in Europeans and in Asians at loci 2q35 and 8p12, and our fine-mapping approach identified new potential causal SNPs at each locus (rs16857609 at locus 2q35, rs7844425 and rs2439304 at locus 8p12) to be prioritized for functional studies.

## MATERIALS AND METHODS

### Study populations and genotyping

We used GWAS data from the EPITHYR consortium that included subjects from seven case-control studies: three were conducted in Metropolitan France (CATHY [28], YOUNG-thyr [29] and E3N [30] studies), two in South Pacific Islands [19, 31], one in Cuba [32] and one in the Gomel region of Belarus, affected by the Chernobyl accident [33]. Study protocols were approved by the local ethic committees and written informed consent was obtained from all participants. Genotyping was conducted using the Infinium Oncoarray-500k Beadchip (Illumina) [34] comprising about 500,000 SNPs to which we incorporated 13,759 additional custom markers involved in TC or thyroid hormones metabolism. Details of the participating studies [19, 28–32, 35] and quality controls (QC) of the genotyping were described in detail previously [14]. QC and ethnicity definition were similar to those described by the OncoArray consortium [34].

After QC, we analysed data from 3,527 Europeans (1,554 cases/1,973 controls) and 649 Asians (301 cases/348 controls). Asian participants came mostly from Polynesia and New-Caledonia so we renamed this group as Oceanians. In order to compare this population to other reference panel, we conducted a principal component analysis (PCA) using the Human Genome Diversity Panel (HGDP) [36]. We observed that Polynesians were closest to East Asian population whereas Melanesians were closest to the Oceanian population (which was from Bougainville). Based on this PCA (Supplementary Figure 6), we separated Oceanian participants into the Melanesian (155 cases/106 controls) and Polynesian subgroups (146 cases/242 controls) in order to reduce the heterogeneity in this population.

### SNP selection

#### At locus 2q35

For the current study, we defined a mapping interval of 162,680bp at 2q35 (218,231,649-218,394,329; NCBI build 37 assembly) that included SNPs in the same linkage disequilibrium (LD) block as the two GWAS SNPs, rs966423 and rs6759952 (*i.e.* SNPs with r² > 0.2) according to the 1000 Genomes Project (phase 1 CEU) [37]. We first catalogued 299 SNPs correlated with one of the two GWAS SNPs at r² > 0.2 and with a minor allele frequency (MAF) greater than 0.02 in Europeans according to the 1000 Genomes Project. We added a set of SNPs to tag the remaining SNPs of the region with MAF > 0.02 and r^2^ > 0.8 by forcing these 299 SNPs and SNPs that were already on the chip. A total of 398 unique tag SNPs were identified of which 226 SNPs were not in the initial design of the chip and were therefore added for genotyping.

As the mapping region was entirely included within the *DIRC3* gene, we chose to extend the region to analyse to 50 kb upstream and downstream from the gene boundaries (572 kb, positions 218,098,746–218,671,316; NCBI build 37 assembly).

Finally, 452 SNPs were genotyped for this region, and 441 of them passed QC in Europeans and in Oceanians (Melanesians and Polynesians). These genotypic data were used to impute the non-genotyped SNPs using IMPUTEv2 [37] and the 1000 Genomes Project (phase 3) as the reference panel [37]. For the imputation in Oceanian individuals, we added the 26 Oceanians from Simons Genome Diversity Panel (SGDP, phase 3) [38] to the 1000 Genomes reference panel. All monomorphic imputed SNPs were excluded and only imputed SNPs with an imputation quality score above 0.3 were kept in the analyses. Hence, the analysis of the 2q35 locus was based on 452 genotyped SNPs and 2,230 imputed SNPs in Europeans, 441 genotyped SNPs and 2053 imputed SNPs in Polynesians, and 441 genotyped SNPs and 1,484 imputed SNPs in Melanesians.

### At locus 8p12

Similarly, at 8p12, the region to be enriched was defined based on the LD block containing rs2439302 (positions 32,158,293–32,641,727; NCBI build 37 assembly). We added 492 SNPs to the design of the chip in this region.

As this region, which included the three previously identified SNPs (rs2439302, rs6996585 and rs2466076), is located within the *NGR1* gene, we extended the analysis to all SNPs located 50kb upstream and downstream from this gene boundaries (1,215 kb; 31,456,820–32,672,558; NCBI build 37 assembly).

Thus, the analysis of the 8p12 locus was based on 552 genotyped SNPs and 5,941 imputed SNPs in Europeans, 497 genotyped SNPs and 5,316 imputed SNPs in Polynesians, and 497 genotyped and 3,728 imputed SNPs in Melanesians.

### Statistical analyses

Imputed SNPs were analysed as expected genotype count (gene dosage). Odds ratios (OR) and 95% confidence interval (CI) testing the single marker association with DTC were calculated using the logistic mixed model (LMM) with the genetic relationship matrix as random effect to account for population structure and cryptic relatedness [39]. All analyses were adjusted for age, sex and study and were stratified by ethnic group. A pooled analysis was also performed with Europeans, Melanesians and Polynesians using the corresponding genetic relationship matrix as a random effect in the LMM. These analyses were performed using Gaston R package [40]. LD between variants was calculated from the imputed data with PLINK (v1.9), using the genotype with a probability higher than 0.9. Otherwise, genotypes were missing for the SNP.

For some variants of interest, we also conducted stratified analysis by study, age group, sex and histology. Heterogeneity of odds ratios across the stratification groups was assessed by using Cochran *Q* test and I² index [41].

For each locus, we first performed conditional analysis in order to test for residual association after accounting for a lead SNP, which permits to make an assumption about the number of independent signals within the region.

### Functional annotation

To assess the possible functional effect of the associated SNPs, several annotation databases with different features were interrogated. We were mainly interested in features involved in regulatory aspects of gene expression including gene promoters, enhancers, transcription factor binding sites (TFBS), untranslated regions (UTR), DNase I hypersensitive sites (DHSs) and histone modifications.

We annotated gene promoter regions and UTR using Gene Elements of GenCode [42], and enhancer elements using FANTOM5 [43] and Roadmap collections [44]. Enhancers are short regions of DNA playing a key role in the transcription of genes by increasing the likelihood that transcription of a particular gene will occur. We also used annotations of super-enhancers available in the catalogue by Hnisz et al. [45]. These elements consist of large clusters of transcriptional enhancers driving expression of genes that define cell identity. We used TFBS database [46] for annotations of TFBS and retrieved annotation of DNase I hypersensitive sites (DHSs) from three sources: Roadmap and maps of human DHS sites as defined by Thurman et al. [47] and by Maurano et al [48]. DHSs are regions of chromatin functionally related to transcriptional activity and are sensitive to cleavage by the DNase I enzyme. Finally, we used chromatin state annotation from the Roadmap database.

To investigate noncoding variants, we also used the following freely available tools, RegulomeDB [49] (https://regulomedb.org/regulome-search/) and Haploreg V4 (http://www.broadinstitute.org/mammals/haploreg). These tools predict whether a given SNP may alter the regulatory protein binding sites, chromatin structure and histone modifications.

In addition, expression Quantitative Trait Locus (eQTL) information was used to perform a colocalization analysis within each genomic region using the Coloc R package [50]. This Bayesian method allows to assess whether two related traits share a causal variant by calculating a posterior probability of causality (PP) for each SNP. For this analysis, we used results of the association test between each SNP and DTC risk and results of the association test between each SNP and gene expression in thyroid tumour cells that are publicly available in the PancanQTL database [17]. PancanQTL obtained the gene expression profiles from The Cancer Genome Atlas (TCGA) (http://cancergenome.nih.gov/abouttcga). In Europeans, among the 2,682 tested variants at locus 2q35, 564 were cis-eQTLs, that is, an eQTL that acts in (locally) to a gene. At locus 8p12, among the 6,493 tested variants, 736 were cis-eQTLs.

## SUPPLEMENTARY MATERIALS





## Abbreviations

DHS: DNase I hypersensitive sites
DTC: differentiated thyroid carcinoma
eQTL: expression quantitative trait locus
FTC: follicular thyroid carcinoma
GWAS: genome-wide association study
LD: linkage disequilibrium
LMM: logistic mixed model
MAF: minor allele frequency
OR: odds ratio
PCA: principal component analysis
PP: posterior probability
PTC: papillary thyroid carcinoma
PY: person-year
QC: quality control
SNP: single nucleotide polymorphism
TC: thyroid cancer
TFBS: transcription factor binding site
TSH: thyroid stimulating hormone
UTR: untranslated region
