# Supplementary material for: Fine–mapping of two differentiated thyroid carcinoma susceptibility loci at 2q35 and 8p12 in Europeans, Melanesians and Polynesians
Source: Oncotarget. 2021 Mar 2;12(5):493–506. doi: 10.18632/oncotarget.27888 (PMC7939525; doi:10.18632/oncotarget.27888)
Supplement: Supplementary file 1 [file oncotarget-12-493-s001.pdf]

Fine–mapping of two differentiated thyroid carcinoma susceptibility loci at 2q35 and 8p12 in Europeans, Melanesians and Polynesians

SUPPLEMENTARY MATERIALS

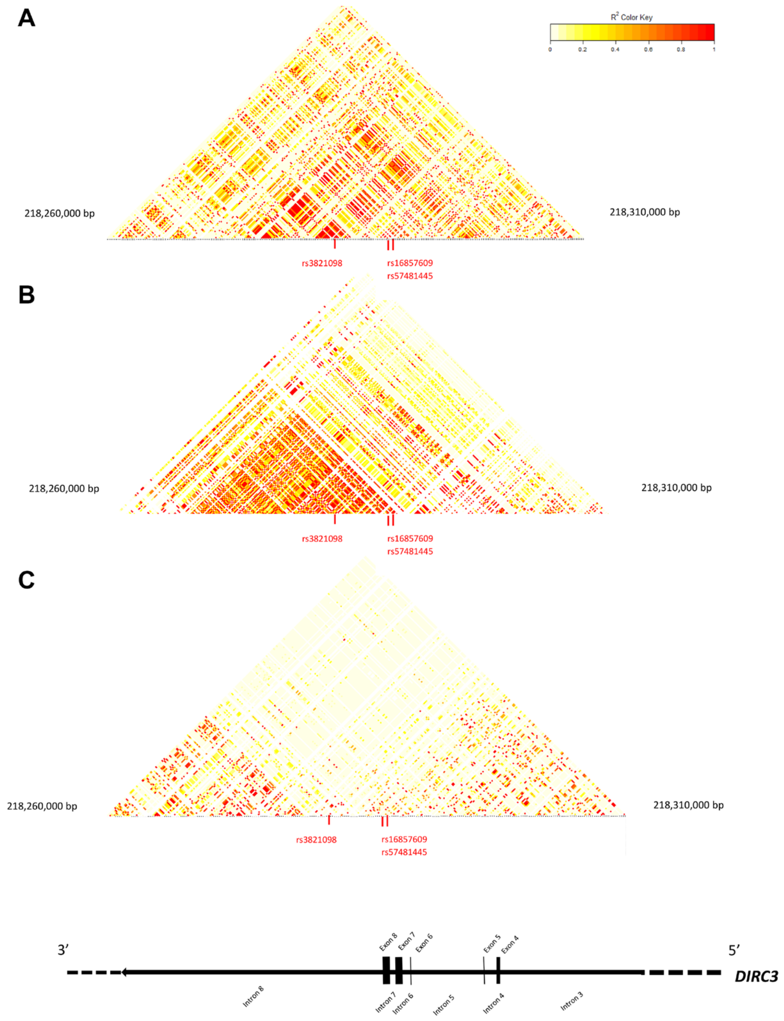

2q35 region (chr2:218,260,000-218,310,000) showing the 3′ end of *DIRC3*. Exons and introns are numbered according to transcript NR_026597.2.

Supplementary Figure 1: Heatmap of correlation between SNPs at locus 2q35 for Europeans (**A**), Melanesians (**B**) and Polynesians (**C**).

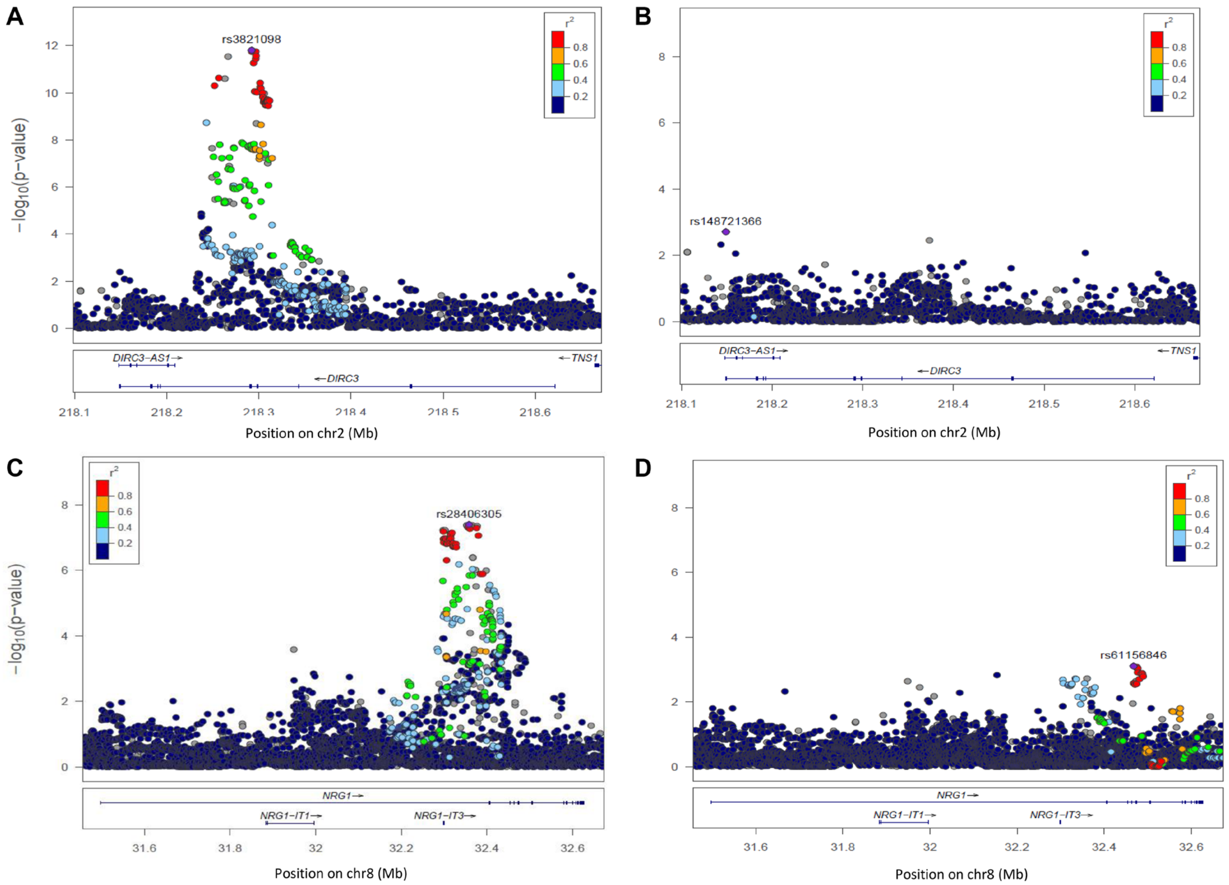

Supplementary Figure 2: Regional plot of single-SNP association and conditional regression at loci 2q35 and 8p12 in the pooled analysis. On Y axis, are the –log10 (*p*-value); on X axis, the position of SNPs and gene on the chromosome. The color of each SNP spot reflects its r² with the most associated SNP (in purple). The correlation between SNP is ranged from high (in red) to low (in blue). Grey dots correspond to missing variant (imputed SNP with no genotype with probability > 0.9 (**A**) Plot of –log10 (*p*-values) of SNP association results at locus 2q35 in the pooled analysis. (**B**) Plot of –log10 (*p*-values) of SNP association results at locus 2q35 after conditioning analysis on SNP rs3821098. (**C**) Plot of –log10 (*p*-values) of SNP association results at locus 8p12 in the pooled analysis. (**D**) Plot of –log10 (*p*-values) of association results at locus 8p12 after conditioning analysis on SNP rs28406305.

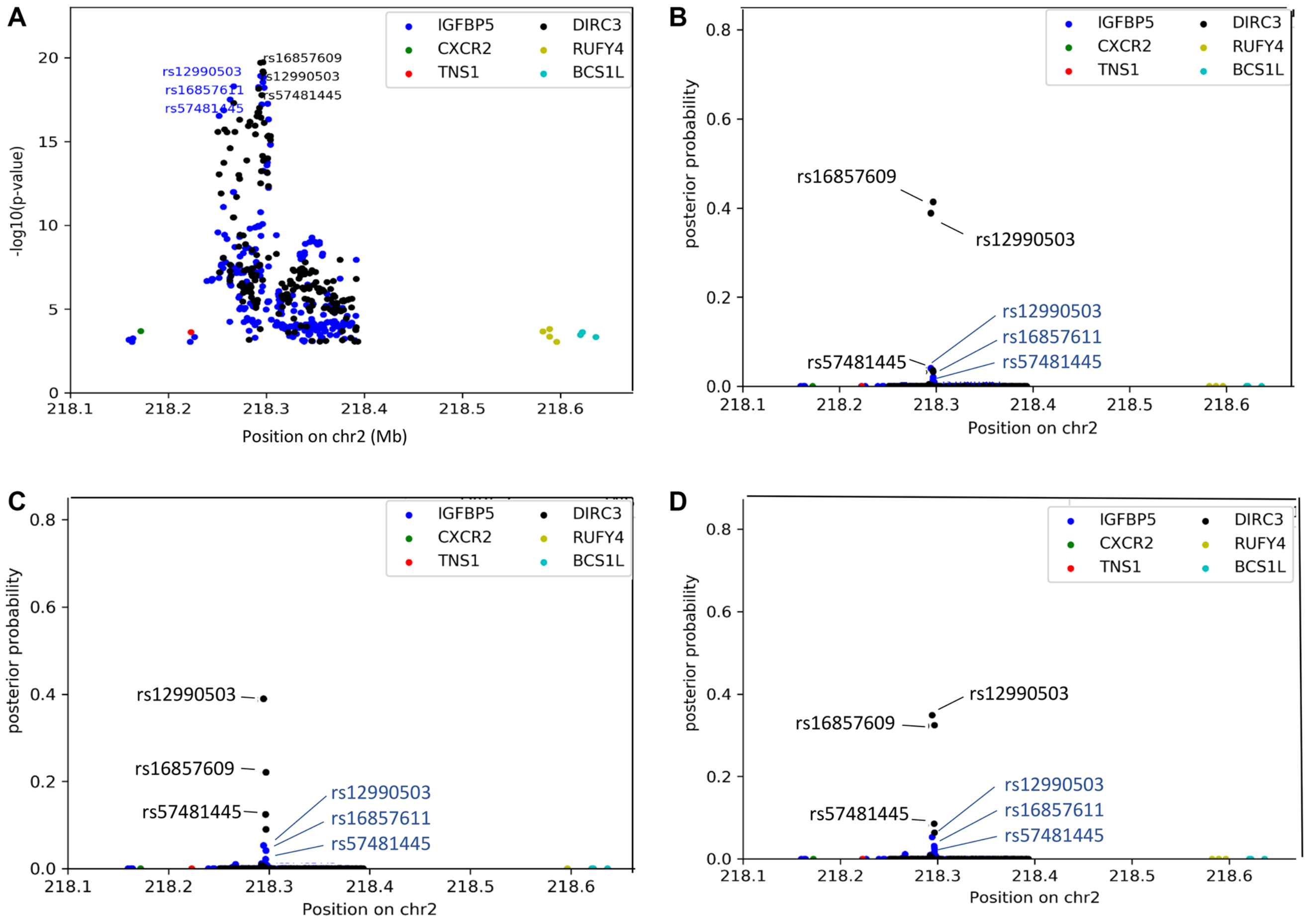

Supplementary Figure 3: (**A**) Plot of –log10 (*p*-values) of association between SNPs and gene expression in thyroid tumor cells. Plot of posterior probabilities for each SNP to be causal obtained in colocalization analyses in (**B**) Europeans, (**C**) Melanesians and (**D**) Polynesians at locus 2q35.

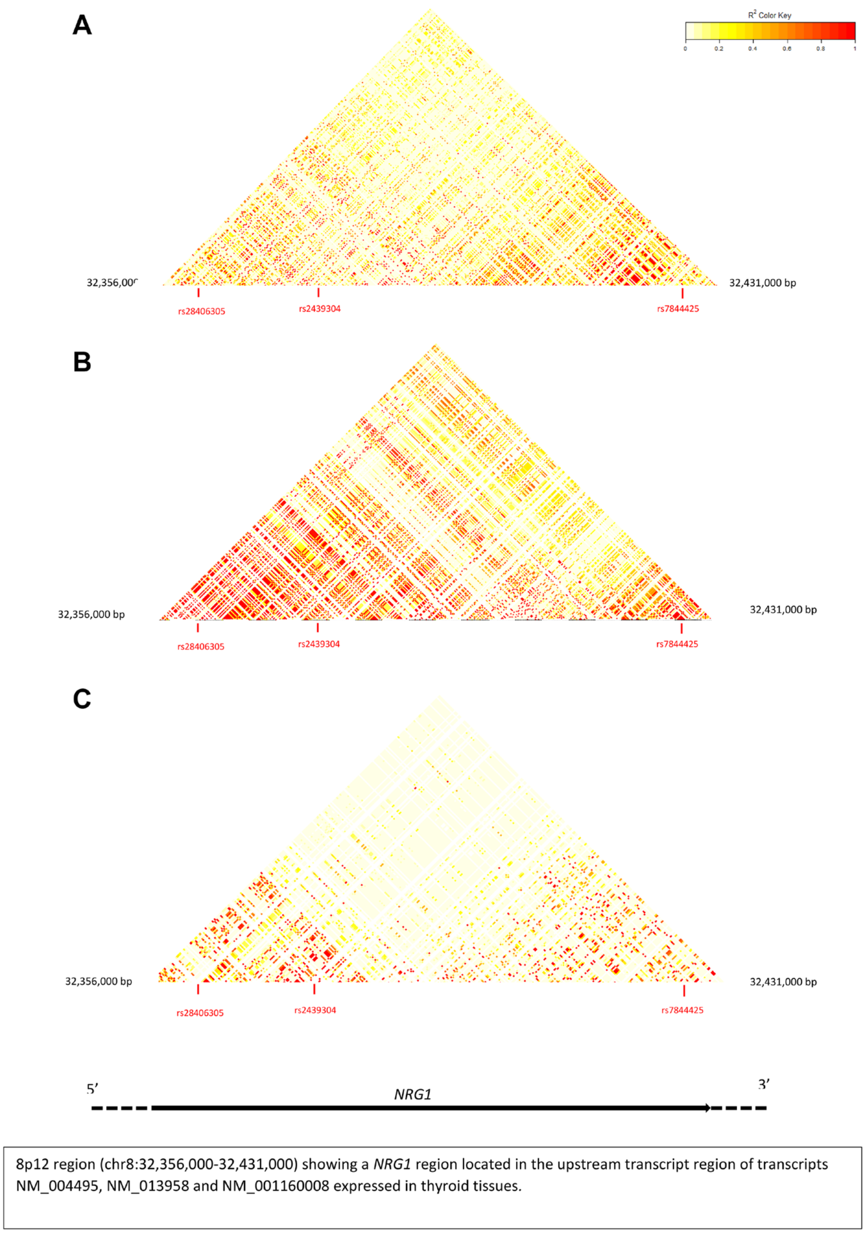

Supplementary Figure 4: Heatmap of correlation between SNPs at locus 8p12 for Europeans (**A**), Melanesians (**B**) and Polynesians (**C**).

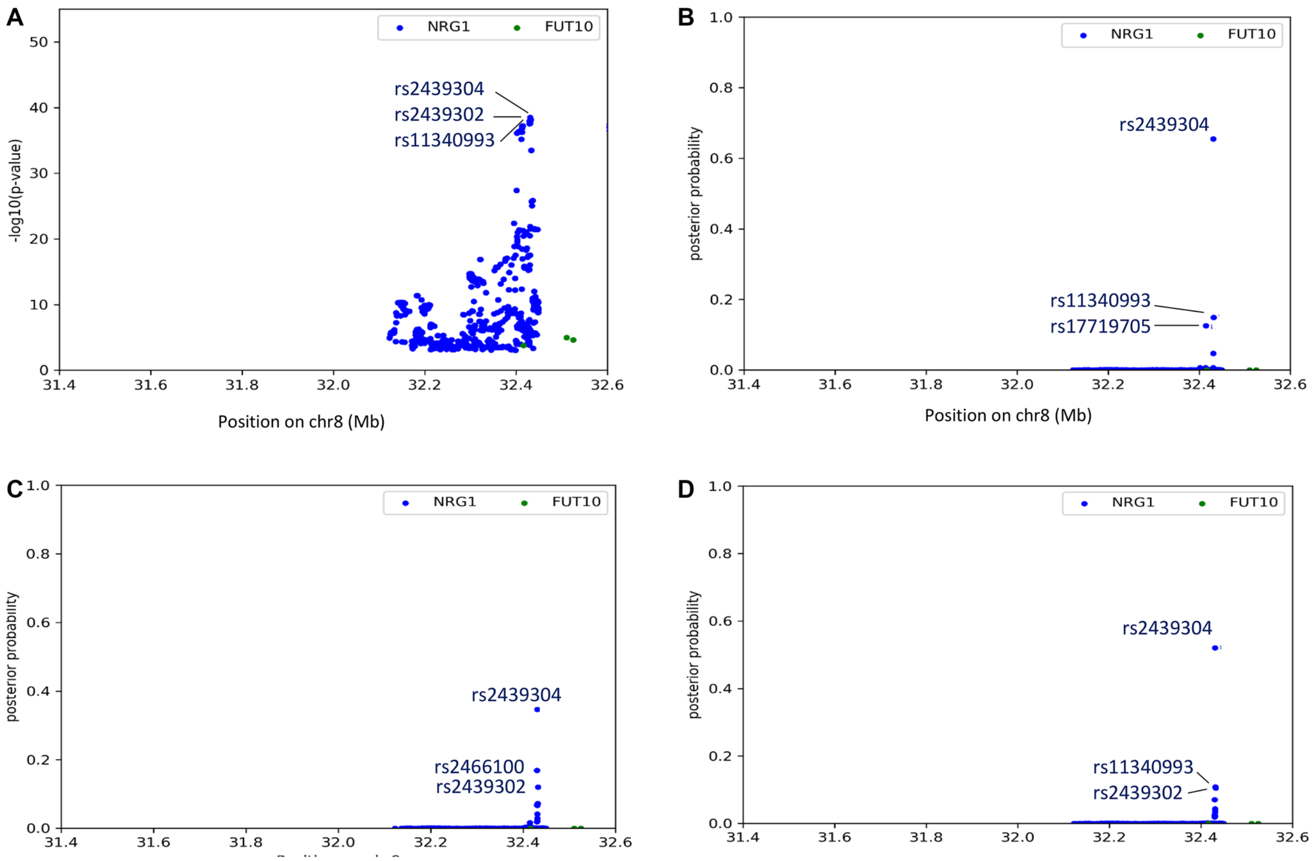

Supplementary Figure 5: (**A**) Plot of –log10 p-values of association between SNPs and gene expression in thyroid tumor cells. Plot of posterior probabilities for each SNP to be causal obtained colocalization analyses in (**B**) Europeans, (**C**) Melanesians and (**D**) Polynesians at locus 8p12.

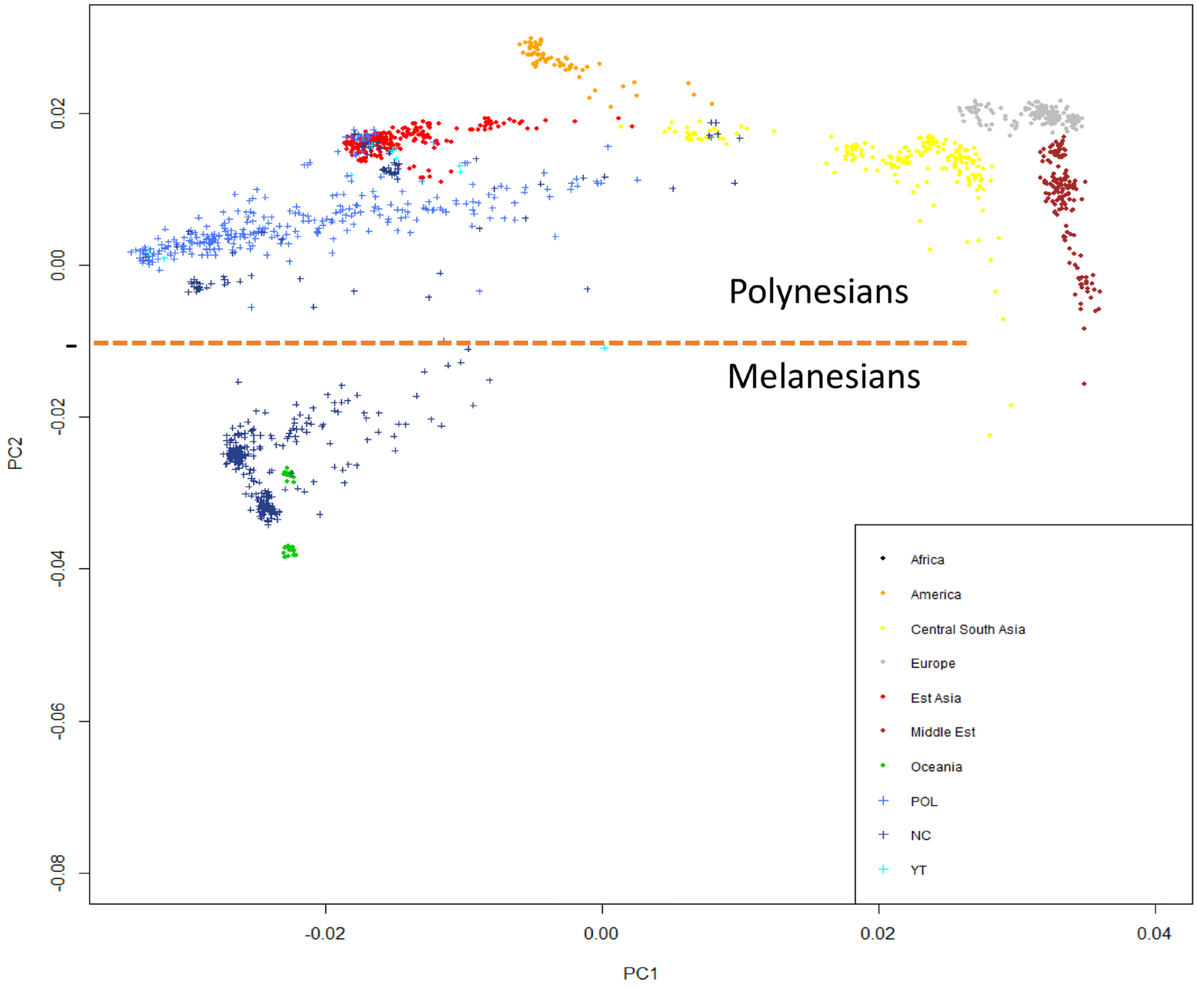

Supplementary Figure 6: Principal component analysis of the Asian population (Oceanians) using the Human Genome Diversity Panel as the reference panel. Legend: POL, subjects from the French Polynesia study; NC, subjects from the New-Caledonia study; YT, subjects from the YOUNG-thyr.

Supplementary Table 1: Number of SNPs analyzed at loci 2q35 and 8p12 by ethnic group

Locus		Europeans (N = 3527)	Polynesians (N = 388)	Melanesians (N = 261)
**2q35**	Genotyped SNPs	452	441	441
	Imputed SNPs	2230	2053	1484
**8p12**	Genotyped SNPs	552	497	497
	Imputed SNPs	5941	5316	3728

Supplementary Table 2: Association results of the 42 SNPs at 2q35 significantly associated with DTC ($p < 5 \times 10^{-08}$) in Europeans by ethnic group. See Supplementary Table 2

Supplementary Table 3: Association results for previously identified GWAS SNP for loci 2q35 and 8p12 in Europeans, Melanesians and Polynesians. See Supplementary Table 3

Supplementary Table 4: Association results of the 37 SNPs most significant SNPs associated with DTC at 8p12 ($p < 1 \times 10^{-07}$) in Europeans, by ethnic group. See Supplementary Table 4

Supplementary Table 5: Effect allele frequency of associated SNPs at loci 2q35 and 8p12 in the studied populations and in the 1000Genomes database. See Supplementary Table 5
