# Supplementary material for: Fine–mapping of two differentiated thyroid carcinoma susceptibility loci at 2q35 and 8p12 in Europeans, Melanesians and Polynesians
Source: Oncotarget. 2021 Mar 2;12(5):493–506. doi: 10.18632/oncotarget.27888 (PMC7939525; doi:10.18632/oncotarget.27888)
Supplement: Supplementary file 2 [file oncotarget-12-493-s002.docx]

| **Supplementary Table 2: Association results of the 42 SNPs at 2q35 significantly associated with DTC (*p* < 5 x 10^-08^) in Europeans by ethnic group.** | | | | | | | | | | | | | | | | | | | | | | | | | | | |  | |  | | |  | | | | |  | | |  | | |  |
| --- | --- | --- | --- | --- | --- | --- | --- | --- | --- | --- | --- | --- | --- | --- | --- | --- | --- | --- | --- | --- | --- | --- | --- | --- | --- | --- | --- | --- | --- | --- | --- | --- | --- | --- | --- | --- | --- | --- | --- | --- | --- | --- | --- | --- |
|  |  |  | |  |  | **Europeans** | | | | | | | | **Polynesians** | | | | | | | | **Melanesians** | | | | | | | **Pooled** | | | | | | | | | | | | | | | |
| **SNP** | **R info^a^** | **Position** | | **A1** | **A2^b^** | **Freq. A1** | | | **Freq. A2** | **OR** | | **p** | | **Freq A1** | | **Freq A2** | | **OR** | **p** | | | **Freq. A1** | **Freq. A2** | | | **OR** | **p** | | **Freq.** | | | **Freq.** | | | | | **OR** | | | **p** | | | | |
|  |  |  |  |  |  |  |  |  |  |  |  |  |  |  |  |  |  |  |  |  |  |  |  |  |  |  |  |  | **A1** | | | **A2** | | | | |  |  |  |  |  |  |  |  |
| rs57481445 |  | 218296374 | | A | G | 0,72 | | | 0,28 | 1,43 | | 8.6x10^-11^ | | 0,18 | | 0,82 | | 1,42 | 0,13 | | | 0,08 | 0,92 | | | 1,05 | 0,88 | | 0,63 | | | 0,37 | | | | | 1,45 | | | 1.8x10^-12^ | | | | |
| rs3821098 |  | 218292141 | | T | C | 0,28 | | | 0,72 | 1,43 | | 9.7x10^-11^ | | 0,83 | | 0,17 | | 1,44 | 0,09 | | | 0,92 | 0,08 | | | 1,10 | 0,77 | | 0,37 | | | 0,63 | | | | | 1,44 | | | 1.6X10^-12^ | | | | |
| rs11693806* | > 0.98 | 218292158 | | C | G | 0,30 | | | 0,70 | 0,70 | | 1.1x10^-10^ | | 0,83 | | 0,17 | | 0,67 | 0,09 | | | 0,92 | 0,08 | | | 0,91 | 0,77 | | 0,38 | | | 0,62 | | | | | 0,69 | | | 1.7x10^-12^ | | | | |
| rs16857611 |  | 218296732 | | C | T | 0,72 | | | 0,28 | 1,42 | | 1.2x10^-10^ | | 0,18 | | 0,82 | | 1,41 | 0,13 | | | 0,08 | 0,92 | | | 1,03 | 0,92 | | 0,63 | | | 0,37 | | | | | 1,44 | | | 2.7x10^-12^ | | | | |
| rs66470855* | > 0.99 | 218266438 | | TG | T | 0,72 | | | 0,28 | 1,42 | | 1.6x10^-10^ | | 0,18 | | 0,82 | | 1,38 | 0,14 | | | 0,08 | 0,92 | | | 1,12 | 0,73 | | 0,63 | | | 0,37 | | | | | 1,44 | | | 2.9x10^-12^ | | | | |
| rs16857609 |  | 218296508 | | C | T | 0,72 | | | 0,28 | 1,42 | | 1.6x10^-10^ | | 0,18 | | 0,82 | | 1,42 | 0,13 | | | 0,08 | 0,92 | | | 1,05 | 0,88 | | 0,63 | | | 0,37 | | | | | 1,44 | | | 3.5x10^-12^ | | | | |
| rs12990503 |  | 218294217 | | C | G | 0,29 | | | 0,71 | 0,67 | | 3.4x10^-10^ | | 0,83 | | 0,17 | | 1,46 | 0,09 | | | 0,92 | 0,08 | | | 1,10 | 0,76 | | 0,38 | | | 0,62 | | | | | 0,70 | | | 5.4x10^-12^ | | | | |
| rs6723847* | > 0.99 | 218256489 | | C | T | 0,69 | | | 0,31 | 1,39 | | 1.1x10^-09^ | | 0,18 | | 0,82 | | 1,37 | 0,15 | | | 0,08 | 0,92 | | | 1,13 | 0,72 | | 0,61 | | | 0,39 | | | | | 1,41 | | | 2.3x10^-11^ | | | | |
| rs5838651* |  | 218262857 | | TG | T | 0,69 | | | 0,31 | 1,38 | | 1.2x10^-09^ | | 0,18 | | 0,82 | | 1,38 | 0,14 | | | 0,08 | 0,92 | | | 1,12 | 0,73 | | 0,60 | | | 0,40 | | | | | 1,41 | | | 2.0x10^-11^ | | | | |
| rs13002451 |  | 218301341 | | A | G | 0,69 | | | 0,31 | 1,38 | | 2.2x10^-09^ | | 0,16 | | 0,84 | | 1,52 | 0,08 | | | 0,05 | 0,95 | | | 0,95 | 0,89 | | 0,60 | | | 0,40 | | | | | 1,41 | | | 3.8X10^-11^ | | | | |
| rs6706673 |  | 218251702 | | G | A | 0,69 | | | 0,31 | 1,38 | | 2.2x10^-09^ | | 0,18 | | 0,82 | | 1,37 | 0,16 | | | 0,08 | 0,92 | | | 1,13 | 0,73 | | 0,61 | | | 0,39 | | | | | 1,40 | | | 5.0x10^-11^ | | | | |
| rs6760809* | > 0.99 | 218305715 | | C | T | 0,70 | | | 0,30 | 1,38 | | 2.5x10^-09^ | | 0,24 | | 0,76 | | 1,38 | 0,11 | | | 0,10 | 0,90 | | | 0,95 | 0,88 | | 0,62 | | | 0,38 | | | | | 1,40 | | | 1.1x10^-10^ | | | | |
| rs4674176 |  | 218301119 | | G | C | 0,31 | | | 0,69 | 0,73 | | 3.5x10^-09^ | | 0,83 | | 0,17 | | 0,65 | 0,07 | | | 0,95 | 0,05 | | | 1,07 | 0,87 | | 0,40 | | | 0,60 | | | | | 0,71 | | | 5.7x10^-11^ | | | | |
| rs13388294 |  | 218295406 | | G | A | 0,70 | | | 0,30 | 1,38 | | 3.8x10^-09^ | | 0,18 | | 0,82 | | 1,42 | 0,13 | | | 0,08 | 0,92 | | | 1,05 | 0,88 | | 0,62 | | | 0,38 | | | | | 1,40 | | | 9.0x10^-11^ | | | | |
| rs79045636* | > 0.99 | 218304448 | | T | TAAAC | 0,69 | | | 0,31 | 1,37 | | 3.9x10^-09^ | | 0,22 | | 0,78 | | 1,36 | 0,12 | | | 0,08 | 0,92 | | | 1,04 | 0,89 | | 0,61 | | | 0,39 | | | | | 1,39 | | | 1.2x10^-10^ | | | | |
| rs372483743* | > 0.99 | 218304440 | | T | TAAAC | 0,69 | | | 0,31 | 1,37 | | 3.9x10^-09^ | | 0,22 | | 0,78 | | 1,36 | 0,12 | | | 0,08 | 0,92 | | | 1,04 | 0,89 | | 0,61 | | | 0,39 | | | | | 1,39 | | | 1.2x10^-10^ | | | | |
| rs2618147 |  | 218302138 | | C | A | 0,69 | | | 0,31 | 1,37 | | 4.1x10^-09^ | | 0,17 | | 0,83 | | 1,53 | 0,07 | | | 0,06 | 0,94 | | | 0,94 | 0,89 | | 0,60 | | | 0,40 | | | | | 1,40 | | | 6.6x10^-11^ | | | | |
| rs2568176* | > 0.97 | 218303958 | | G | A | 0,69 | | | 0,31 | 1,37 | | 4.2x10^-09^ | | 0,21 | | 0,79 | | 1,40 | 0,11 | | | 0,08 | 0,92 | | | 1,05 | 0,88 | | 0,61 | | | 0,39 | | | | | 1,39 | | | 1.0x10^-10^ | | | | |
| rs2618148 |  | 218304106 | | C | T | 0,69 | | | 0,31 | 1,37 | | 4.5x10^-09^ | | 0,22 | | 0,78 | | 1,34 | 0,14 | | | 0,08 | 0,92 | | | 1,06 | 0,86 | | 0,61 | | | 0,39 | | | | | 1,39 | | | 1.5x10^-10^ | | | | |
| rs12988242* | > 0.98 | 218306194 | | G | A | 0,69 | | | 0,31 | 1,37 | | 5.1x10^-09^ | | 0,22 | | 0,78 | | 1,36 | 0,13 | | | 0,08 | 0,92 | | | 1,04 | 0,91 | | 0,61 | | | 0,39 | | | | | 1,39 | | | 1.9x10^-10^ | | | | |
| rs11680689 |  | 218297700 | | C | G | 0,31 | | | 0,69 | 0,73 | | 5.2x10^-09^ | | 0,83 | | 0,17 | | 0,65 | 0,07 | | | 0,94 | 0,06 | | | 1,10 | 0,82 | | 0,40 | | | 0,60 | | | | | 0,72 | | | 9.5x10^-11^ | | | | |
| rs35063240* | > 0.99 | 218306947 | | T | TC | 0,69 | | | 0,31 | 1,37 | | 5.3x10^-09^ | | 0,22 | | 0,78 | | 1,36 | 0,13 | | | 0,08 | 0,92 | | | 0,99 | 0,97 | | 0,61 | | | 0,39 | | | | | 1,38 | | | 2.2x10^-10^ | | | | |
| rs12694417* | > 0.99 | 218306089 | | C | T | 0,69 | | | 0,31 | 1,37 | | 5.3x10^-09^ | | 0,22 | | 0,78 | | 1,35 | 0,13 | | | 0,08 | 0,92 | | | 1,04 | 0,91 | | 0,61 | | | 0,39 | | | | | 1,38 | | | 2.0x10^-10^ | | | | |
| rs974406* | > 0.99 | 218308038 | | G | C | 0,69 | | | 0,31 | 1,37 | | 5.4x10^-09^ | | 0,22 | | 0,78 | | 1,35 | 0,13 | | | 0,08 | 0,92 | | | 0,94 | 0,84 | | 0,61 | | | 0,39 | | | | | 1,38 | | | 2.5x10^-10^ | | | | |
| rs2045933 |  | 218307456 | | T | A | 0,69 | | | 0,31 | 1,37 | | 5.4x10^-09^ | | 0,22 | | 0,78 | | 1,36 | 0,13 | | | 0,08 | 0,92 | | | 0,93 | 0,82 | | 0,61 | | | 0,39 | | | | | 1,38 | | | 2.6x10^-10^ | | | | |
| rs2045932 |  | 218307223 | | T | C | 0,69 | | | 0,31 | 1,37 | | 5.6x10^-09^ | | 0,22 | | 0,78 | | 1,33 | 0,16 | | | 0,08 | 0,92 | | | 0,93 | 0,83 | | 0,61 | | | 0,39 | | | | | 1,38 | | | 3.3x10^-10^ | | | | |
| rs6712801 |  | 218308501 | | G | A | 0,69 | | | 0,31 | 1,37 | | 5.7x10^-09^ | | 0,22 | | 0,78 | | 1,32 | 0,17 | | | 0,08 | 0,92 | | | 1,06 | 0,86 | | 0,61 | | | 0,39 | | | | | 1,38 | | | 2.3x10^-10^ | | | | |
| rs4672831 |  | 218309020 | | G | A | 0,69 | | | 0,31 | 1,37 | | 5.7x10^-09^ | | 0,22 | | 0,78 | | 1,36 | 0,13 | | | 0,08 | 0,92 | | | 1,06 | 0,86 | | 0,61 | | | 0,39 | | | | | 1,39 | | | 2.0x10^-10^ | | | | |
| rs1478596 |  | 218309936 | | G | C | 0,69 | | | 0,31 | 1,37 | | 5.7x10^-09^ | | 0,27 | | 0,73 | | 1,33 | 0,14 | | | 0,11 | 0,89 | | | 1,12 | 0,72 | | 0,62 | | | 0,38 | | | | | 1,38 | | | 3.4x10^-10^ | | | | |
| rs4142171 |  | 218309740 | | T | G | 0,69 | | | 0,31 | 1,37 | | 5.8x10^-09^ | | 0,22 | | 0,78 | | 1,36 | 0,13 | | | 0,08 | 0,92 | | | 1,06 | 0,86 | | 0,61 | | | 0,39 | | | | | 1,38 | | | 2.0x10^-10^ | | | | |
| rs4672832 |  | 218309228 | | C | A | 0,69 | | | 0,31 | 1,37 | | 5.8x10^-09^ | | 0,22 | | 0,78 | | 1,36 | 0,13 | | | 0,08 | 0,92 | | | 1,06 | 0,86 | | 0,61 | | | 0,39 | | | | | 1,38 | | | 2.0x10^-10^ | | | | |
| rs73069129* | > 0.99 | 218305901 | | A | C | 0,69 | | | 0,31 | 1,36 | | 5.8x10^-09^ | | 0,22 | | 0,78 | | 1,35 | 0,13 | | | 0,08 | 0,92 | | | 1,04 | 0,91 | | 0,61 | | | 0,39 | | | | | 1,38 | | | 2.1x10^-10^ | | | | |
| rs1318847 |  | 218307695 | | C | T | 0,69 | | | 0,31 | 1,37 | | 5.9x10^-09^ | | 0,22 | | 0,78 | | 1,36 | 0,13 | | | 0,08 | 0,92 | | | 0,93 | 0,83 | | 0,61 | | | 0,39 | | | | | 1,38 | | | 2.9x10^-10^ | | | | |
| rs66934611* | > 0.99 | 218307754 | | T | TTGAG | 0,69 | | | 0,31 | 1,37 | | 6.0x10^-09^ | | 0,22 | | 0,78 | | 1,35 | 0,13 | | | 0,08 | 0,92 | | | 0,93 | 0,82 | | 0,61 | | | 0,39 | | | | | 1,38 | | | 2.9x10^-10^ | | | | |
| rs6729012* | > 0.99 | 218305393 | | A | C | 0,69 | | | 0,31 | 1,37 | | 6.0x10^-09^ | | 0,22 | | 0,78 | | 1,35 | 0,13 | | | 0,08 | 0,92 | | | 1,04 | 0,91 | | 0,61 | | | 0,39 | | | | | 1,38 | | | 2.2x10^-10^ | | | | |
| rs4674177 |  | 218309221 | | C | G | 0,69 | | | 0,31 | 1,37 | | 6.0x10^-09^ | | 0,22 | | 0,78 | | 1,35 | 0,13 | | | 0,08 | 0,92 | | | 1,07 | 0,83 | | 0,61 | | | 0,39 | | | | | 1,38 | | | 2.0x10^-10^ | | | | |
| rs2618150 |  | 218311446 | | A | G | 0,69 | | | 0,31 | 1,36 | | 6.3x10^-09^ | | 0,22 | | 0,78 | | 1,36 | 0,13 | | | 0,08 | 0,92 | | | 1,06 | 0,86 | | 0,61 | | | 0,39 | | | | | 1,38 | | | 2.2x10^-10^ | | | | |
| rs4674178* | > 0.99 | 218309258 | | T | C | 0,69 | | | 0,31 | 1,37 | | 6.6x10^-09^ | | 0,22 | | 0,78 | | 1,35 | 0,13 | | | 0,08 | 0,92 | | | 1,07 | 0,83 | | 0,61 | | | 0,39 | | | | | 1,38 | | | 2.2x10^-10^ | | | | |
| rs974405 |  | 218307910 | | T | C | 0,69 | | | 0,31 | 1,36 | | 6.7x10^-09^ | | 0,22 | | 0,78 | | 1,36 | 0,13 | | | 0,08 | 0,92 | | | 0,93 | 0,83 | | 0,61 | | | 0,39 | | | | | 1,38 | | | 3.2x10^-10^ | | | | |
| rs1478595 |  | 218309899 | | T | G | 0,69 | | | 0,31 | 1,36 | | 6.7x10^-09^ | | 0,22 | | 0,78 | | 1,36 | 0,13 | | | 0,08 | 0,92 | | | 1,06 | 0,86 | | 0,61 | | | 0,39 | | | | | 1,38 | | | 2.4x10^-10^ | | | | |
| rs6715218 |  | 218305064 | | T | C | 0,69 | | | 0,31 | 1,36 | | 7.1x10^-09^ | | 0,22 | | 0,78 | | 1,36 | 0,13 | | | 0,08 | 0,92 | | | 1,06 | 0,86 | | 0,61 | | | 0,39 | | | | | 1,38 | | | 2.5x10^-10^ | | | | |
| rs10211305 |  | 218309267 | | T | A | 0,69 | | | 0,31 | 1,36 | | 7.2x10^-09^ | | 0,22 | | 0,78 | | 1,35 | 0,13 | | | 0,08 | 0,92 | | | 1,07 | 0,83 | | 0,61 | | | 0,39 | | | | | 1,38 | | | 2.5x10^-10^ | | | | |
| * imputed SNPs | |  |  | | | |  |  | | |  | |  | |  | |  | | |  |  | | |  |  | | |  | | |  | | |  |  |  | | |  | | |  |  |  |
| ^a^ Lowest quality score of imputation among all populations | | | | | | | | | | |  | |  | |  | |  | | |  |  | | |  |  | | |  | | |  | | |  |  |  | | |  | | |  |  |  |
| ^b^ Effect allele | | |  | | | |  |  | | |  | |  | |  | |  | | |  |  | | |  |  | | |  | | |  | | |  |  |  | | |  | | |  |  |  |

**Supplementary Table 3: Association results for previously identified GWAS SNP for loci 2q35 and 8p12 in Europeans, Melanesians and Polynesians.**

| **Locus** | **SNP** | **Position (hg19)** | **OA** | **EA** | **Previous GWAS** | | | | **EPITHYR** | | | | | | | | |
| --- | --- | --- | --- | --- | --- | --- | --- | --- | --- | --- | --- | --- | --- | --- | --- | --- | --- |
|  |  |  |  |  |  |  |  |  | **Europeans** | | | **Polynesians** | | | **Melanesians** | | |
|  |  |  |  |  | **Study (year), country** | **EAF** | **OR** | **p** | **EAF** | **OR** | **p** | **EAF** | **OR** | **p** | **EAF** | **OR** | **p** |
| **2q35** | rs1549738 | 218,118,722 | G | A | Son (2017), Korea | 0.61 | 1.28 | 2.9x10^-4^ | 0.14 | 1.03 | 0.70 | 0.86 | 0.99 | 0.97 | 0.94 | 0.36 | 0.03 |
|  | rs6759952 | 218,271,719 | C | T | Kohler(2013), Italy | 0.42 | 1.32 | 9.8x10^-6^ | 0.44 | 1.26 | 2.9x10^-10^ | 0.90 | 1.16 | 0.58 | 0.98 | 0.35 | 0.20 |
|  | rs11693806 | 218,292,158 | G | C | Gudmundsson (2017), Iceland | 0.28 | 1.49 | 3.6x10^-14^ | 0.30 | 1.43 | 1.1x10^-10^ | 0.83 | 1.49 | 0.09 | 0.92 | 1.10 | 0.77 |
|  | rs12990503 | 218,294,217 | C | G | Son (2017), Korea | 0.68 | 1.32 | 1.8x10^-4^ | 0.29 | 0.67 | 3.0x10^-10^ | 0.83 | 1.46 | 0.09 | 0.92 | 1.10 | 0.75 |
|  | rs1382435 | 218,296,003 | C | T | Kohler (2013), Italy | - | 1.51 | 7.2x10^-6^ | 0.35 | 1.32 | 1.2x10^-7^ | 0.88 | 1.22 | 0.43 | 0.98 | 0.27 | 0.10 |
|  | rs966423 | 218,310,340 | T | C | Gudmundsson (2012), Iceland | 0.49 | 1.26 | 3.8x10^-4^ | 0.43 | 1.27 | 2.6x10^-6^ | 0.89 | 1.13 | 0.64 | 0.98 | 0.35 | 0.20 |
|  |  |  |  |  | Gudmundsson (2017), Iceland | 0.44 | 1.32 | 3.2x10^-8^ |  |  |  |  |  |  |  |  |  |
|  |  |  |  |  | Son (2017),Korea | - | 1.24 | 8.1x10^-3^ |  |  |  |  |  |  |  |  |  |
| **8p12** | rs12542743 | 32,318,355 | T | C | Son (2017), Korea | 0.25 | 1.42 | 1.1x10^-6^ | 0.45 | 1.22 | 5.9x10^-5^ | 0.63 | 1.05 | 0.77 | 0.65 | 1.26 | 0.22 |
|  | rs6996585 | 32,400,803 | A | G | Son (2017), Korea | 0.30 | 1.48 | 1.2x10^-7^ | 0.44 | 1.21 | 5.9x10^-8^ | 0.54 | 1.09 | 0.57 | 0.63 | 1.16 | 0.42 |
|  | rs2466076 | 32,432,796 | T | G | Gudmundsson (2017), Iceland | 0.21 | 1.32 | 1.5x10^-17^ | 0.48 | 1.21 | 1.0x10^-4^ | 0.31 | 1.10 | 0.56 | 0.40 | 1.05 | 0.79 |
|  | rs2439302 | 32,432,369 | C | G | Gudmundsson (2012), Iceland | 0.45 | 1.41 | 1.3x10^-6^ | 0.47 | 1.20 | 2.6x10^-4^ | 0.30 | 1.10 | 0.55 | 0.40 | 1.05 | 0.79 |
|  |  |  |  |  | Gudmundsson (2017), Iceland | 0.46 | 1.33 | 6.0x10^-9^ |  |  |  |  |  |  |  |  |  |
|  |  |  |  |  | Son (2017), Korea | 0.27 | 1.37 | 1.4x10^-9^ |  |  |  |  |  |  |  |  |  |

OA: other allele; EA: effect allele, EAF: effect allele frequency, OR: odds ratio, p: p-value

| **Supplementary Table 4: Association results of the 37 SNPs most significant SNPs associated with DTC at 8p12 (*p*<1x10^-07^) in Europeans, by ethnic group.** | | | | | | | | | | | | | | | | | | | | | | | | | | | | | |  |  |  |  |  |  |  |  |
| --- | --- | --- | --- | --- | --- | --- | --- | --- | --- | --- | --- | --- | --- | --- | --- | --- | --- | --- | --- | --- | --- | --- | --- | --- | --- | --- | --- | --- | --- | --- | --- | --- | --- | --- | --- | --- | --- |
|  |  |  |  | |  | **Europeans** | | | | | | **Polynesians** | | | | | **Melanesians** | | | | | | | **Pooled** | | | | | | |  |  |  |  |  |  |  |
| **SNP** | **R info^a^** | **Position** | **A1** | | **A2^b^** | **Freq. A1** | **Freq. A2** | | | **OR** | **p** | **Freq A1** | **Freq A2** | **OR** | | **p** | **Freq. A1** | | **Freq. A2** | | **OR** | **p** | | **Freq, A1** | **Freq, A2** | **OR** | | **p** | | |  |  |  |  |  |  |  |
|  |  |  |  |  |  |  |  |  |  |  |  |  |  |  |  |  |  |  |  |  |  |  |  |  |  |  |  |  |  |  |  |  |  |  |  |  |  |
| rs147012828* | > 0.99 | 32297390 | A | | AAT | 0,65 | 0,35 | | | 1,32 | 1.0x10^-07^ | 0,77 | 0,23 | 1,20 | | 0,31 | 0,80 | | 0,20 | | 1,40 | 0,15 | | 0,67 | 0,33 | 1,30 | | 6.1x10^-08^ | | |  |  |  |  |  |  |  |
| rs4129579 |  | 32297851 | G | | A | 0,65 | 0,35 | | | 1,32 | 1.0x10^-07^ | 0,77 | 0,23 | 1,18 | | 0,34 | 0,80 | | 0,20 | | 1,40 | 0,14 | | 0,67 | 0,33 | 1,30 | | 6.5x10^-08^ | | |  |  |  |  |  |  |  |
| rs1579033* | > 0.99 | 32298180 | G | | C | 0,65 | 0,35 | | | 1,31 | 1.7x10^-07^ | 0,77 | 0,23 | 1,18 | | 0,35 | 0,80 | | 0,20 | | 1,40 | 0,14 | | 0,67 | 0,33 | 1,30 | | 1.1x10^-07^ | | |  |  |  |  |  |  |  |
| rs698166 |  | 32298476 | T | | C | 0,65 | 0,35 | | | 1,31 | 2.2x10^-07^ | 0,77 | 0,23 | 1,18 | | 0,36 | 0,80 | | 0,20 | | 1,40 | 0,14 | | 0,67 | 0,33 | 1,30 | | 1.4x10^-07^ | | |  |  |  |  |  |  |  |
| rs6468103* | > 0.99 | 32301324 | C | | T | 0,65 | 0,35 | | | 1,31 | 1.7x10^-07^ | 0,77 | 0,23 | 1,21 | | 0,28 | 0,81 | | 0,19 | | 1,37 | 0,18 | | 0,67 | 0,33 | 1,30 | | 1.0x10^-07^ | | |  |  |  |  |  |  |  |
| rs150338438* | > 0.99 | 32301542 | TTTCC | | T | 0,65 | 0,35 | | | 1,32 | 1.2x10^-07^ | 0,77 | 0,23 | 1,22 | | 0,27 | 0,81 | | 0,19 | | 1,38 | 0,17 | | 0,67 | 0,33 | 1,31 | | 5.9x10^-08^ | | |  |  |  |  |  |  |  |
| rs6994625 |  | 32303643 | C | | T | 0,65 | 0,35 | | | 1,31 | 1.7x10^-07^ | 0,77 | 0,23 | 1,20 | | 0,31 | 0,81 | | 0,19 | | 1,37 | 0,18 | | 0,67 | 0,33 | 1,30 | | 1.1x10^-07^ | | |  |  |  |  |  |  |  |
| rs13439435 |  | 32305410 | A | | T | 0,65 | 0,35 | | | 1,31 | 2.4x10^-07^ | 0,77 | 0,23 | 1,20 | | 0,31 | 0,81 | | 0,19 | | 1,37 | 0,18 | | 0,67 | 0,33 | 1,29 | | 1.5x10^-07^ | | |  |  |  |  |  |  |  |
| rs28707398 |  | 32305429 | G | | T | 0,66 | 0,34 | | | 1,29 | 9.8x10^-07^ | 0,77 | 0,23 | 1,22 | | 0,26 | 0,81 | | 0,19 | | 1,37 | 0,18 | | 0,68 | 0,32 | 1,28 | | 5.0x10^-07^ | | |  |  |  |  |  |  |  |
| rs6992352* | > 0.99 | 32306674 | A | | G | 0,65 | 0,35 | | | 1,31 | 2.3x10^-07^ | 0,77 | 0,23 | 1,20 | | 0,31 | 0,81 | | 0,19 | | 1,37 | 0,18 | | 0,67 | 0,33 | 1,30 | | 1.5x10^-07^ | | |  |  |  |  |  |  |  |
| rs10098640 |  | 32309898 | G | | A | 0,65 | 0,35 | | | 1,32 | 1.5x10^-07^ | 0,77 | 0,23 | 1,19 | | 0,34 | 0,81 | | 0,19 | | 1,32 | 0,23 | | 0,67 | 0,33 | 1,30 | | 1.3x10^-07^ | | |  |  |  |  |  |  |  |
| rs13439816 |  | 32312485 | G | | A | 0,65 | 0,35 | | | 1,32 | 1.5x10^-07^ | 0,77 | 0,23 | 1,19 | | 0,34 | 0,81 | | 0,19 | | 1,37 | 0,18 | | 0,67 | 0,33 | 1,30 | | 1.1x10^-07^ | | |  |  |  |  |  |  |  |
| rs1010093* | > 0.99 | 32312962 | T | | C | 0,65 | 0,35 | | | 1,31 | 1.6x10^-07^ | 0,77 | 0,23 | 1,19 | | 0,34 | 0,81 | | 0,19 | | 1,37 | 0,18 | | 0,67 | 0,33 | 1,30 | | 1.2x10^-07^ | | |  |  |  |  |  |  |  |
| rs6981184 |  | 32313905 | G | | A | 0,65 | 0,35 | | | 1,31 | 2.0x10^-07^ | 0,77 | 0,23 | 1,19 | | 0,34 | 0,81 | | 0,19 | | 1,37 | 0,18 | | 0,67 | 0,33 | 1,29 | | 1.5x10^-07^ | | |  |  |  |  |  |  |  |
| rs59332083* | > 0.99 | 32314553 | C | | G | 0,65 | 0,35 | | | 1,32 | 1.2x10^-07^ | 0,77 | 0,23 | 1,20 | | 0,30 | 0,81 | | 0,19 | | 1,37 | 0,18 | | 0,67 | 0,33 | 1,30 | | 7.7x10^-08^ | | |  |  |  |  |  |  |  |
| rs16879430 |  | 32314818 | G | | A | 0,65 | 0,35 | | | 1,32 | 1.1x10^-07^ | 0,77 | 0,23 | 1,19 | | 0,34 | 0,81 | | 0,19 | | 1,37 | 0,18 | | 0,67 | 0,33 | 1,30 | | 8.5x10^-08^ | | |  |  |  |  |  |  |  |
| rs1133207* | > 0.96 | 32315432 | CT | | C | 0,64 | 0,36 | | | 1,32 | 9.9x10^-08^ | 0,76 | 0,24 | 1,17 | | 0,37 | 0,81 | | 0,19 | | 1,37 | 0,17 | | 0,67 | 0,33 | 1,30 | | 8.5x10^-08^ | | |  |  |  |  |  |  |  |
| rs7012019* | > 0.95 | 32315490 | A | | G | 0,65 | 0,35 | | | 1,32 | 1.4x10^-07^ | 0,77 | 0,23 | 1,19 | | 0,34 | 0,81 | | 0,19 | | 1,37 | 0,18 | | 0,67 | 0,33 | 1,30 | | 1.1x10^-07^ | | |  |  |  |  |  |  |  |
| rs17716295 |  | 32317917 | C | | A | 0,65 | 0,35 | | | 1,32 | 1.0x10^-07^ | 0,77 | 0,23 | 1,20 | | 0,31 | 0,81 | | 0,19 | | 1,37 | 0,18 | | 0,67 | 0,33 | 1,30 | | 7.1x10^-08^ | | |  |  |  |  |  |  |  |
| rs12056349 |  | 32319473 | G | | A | 0,65 | 0,35 | | | 1,31 | 1.8x10^-07^ | 0,77 | 0,23 | 1,19 | | 0,34 | 0,81 | | 0,19 | | 1,35 | 0,20 | | 0,67 | 0,33 | 1,29 | | 1.5x10^-07^ | | |  |  |  |  |  |  |  |
| rs11997114 |  | 32319958 | T | | C | 0,65 | 0,35 | | | 1,31 | 1.8x10^-07^ | 0,77 | 0,23 | 1,19 | | 0,34 | 0,81 | | 0,19 | | 1,35 | 0,20 | | 0,67 | 0,33 | 1,29 | | 1.4x10^-07^ | | |  |  |  |  |  |  |  |
| rs12056398 |  | 32320729 | G | | C | 0,65 | 0,35 | | | 1,31 | 2.4x10^-07^ | 0,77 | 0,23 | 1,19 | | 0,34 | 0,81 | | 0,19 | | 1,35 | 0,20 | | 0,67 | 0,33 | 1,29 | | 1.9x10^-07^ | | |  |  |  |  |  |  |  |
| rs12056895 |  | 32321107 | A | | G | 0,65 | 0,35 | | | 1,31 | 2.1x10^-07^ | 0,77 | 0,23 | 1,19 | | 0,34 | 0,81 | | 0,19 | | 1,30 | 0,26 | | 0,67 | 0,33 | 1,29 | | 1.9x10^-07^ | | |  |  |  |  |  |  |  |
| rs1205672* | > 0.99 | 32321170 | C | | T | 0,65 | 0,35 | | | 1,31 | 2.2x10^-07^ | 0,77 | 0,23 | 1,19 | | 0,34 | 0,81 | | 0,19 | | 1,34 | 0,22 | | 0,67 | 0,33 | 1,29 | | 1.8x10^-07^ | | |  |  |  |  |  |  |  |
| rs10096770 |  | 32325220 | A | | G | 0,65 | 0,35 | | | 1,31 | 1.8x10^-07^ | 0,76 | 0,24 | 1,17 | | 0,37 | 0,81 | | 0,19 | | 1,30 | 0,26 | | 0,67 | 0,33 | 1,29 | | 1.9x10^-07^ | | |  |  |  |  |  |  |  |
| rs7821785* | > 0.97 | 32327757 | C | | T | 0,65 | 0,35 | | | 1,31 | 1.7x10^-07^ | 0,76 | 0,24 | 1,18 | | 0,36 | 0,82 | | 0,18 | | 1,37 | 0,19 | | 0,67 | 0,33 | 1,29 | | 1.5x10^-07^ | | |  |  |  |  |  |  |  |
| rs7821944* | > 0.97 | 32327879 | C | | A | 0,65 | 0,35 | | | 1,31 | 2.1x10^-07^ | 0,76 | 0,24 | 1,17 | | 0,38 | 0,82 | | 0,18 | | 1,37 | 0,19 | | 0,67 | 0,33 | 1,29 | | 1.9x10^-07^ | | |  |  |  |  |  |  |  |
| rs34757605* | > 0.98 | 32352365 | CCT | | C | 0,65 | 0,35 | | | 1,33 | 6.4x10^-08^ | 0,77 | 0,23 | 1,20 | | 0,31 | 0,83 | | 0,17 | | 1,40 | 0,17 | | 0,67 | 0,33 | 1,31 | | 4.3x10^-08^ | | |  |  |  |  |  |  |  |
| rs11506112 |  | 32355804 | G | | C | 0,65 | 0,35 | | | 1,32 | 7.3x10^-08^ | 0,77 | 0,23 | 1,20 | | 0,30 | 0,83 | | 0,17 | | 1,40 | 0,17 | | 0,67 | 0,33 | 1,31 | | 4.9x10^-08^ | | |  |  |  |  |  |  |  |
| rs10808327* | > 0.99 | 32356018 | T | | C | 0,65 | 0,35 | | | 1,32 | 7.4x10^-08^ | 0,77 | 0,23 | 1,20 | | 0,30 | 0,83 | | 0,17 | | 1,40 | 0,17 | | 0,67 | 0,33 | 1,31 | | 5.0x10^-08^ | | |  |  |  |  |  |  |  |
| rs28406305 |  | 32357923 | C | | T | 0,65 | 0,35 | | | 1,33 | 5.9x10^-08^ | 0,77 | 0,23 | 1,20 | | 0,31 | 0,83 | | 0,17 | | 1,40 | 0,17 | | 0,67 | 0,33 | 1,31 | | 4.1x10^-08^ | | |  |  |  |  |  |  |  |
| rs28570331* | > 0.99 | 32359701 | C | | T | 0,65 | 0,35 | | | 1,32 | 8.0x10^-08^ | 0,77 | 0,23 | 1,20 | | 0,30 | 0,83 | | 0,17 | | 1,40 | 0,17 | | 0,67 | 0,33 | 1,31 | | 5.4x10^-08^ | | |  |  |  |  |  |  |  |
| rs14245047* | > 0.98 | 32365296 | CT | | C | 0,65 | 0,35 | | | 1,33 | 6.2x10^-08^ | 0,77 | 0,23 | 1,20 | | 0,30 | 0,83 | | 0,17 | | 1,40 | 0,17 | | 0,67 | 0,33 | 1,31 | | 4.2x10^-08^ | | |  |  |  |  |  |  |  |
| rs7844425 |  | 32375617 | T | | G | 0,66 | 0,34 | | | 1,32 | 7.6x10^-08^ | 0,77 | 0,23 | 1,20 | | 0,31 | 0,83 | | 0,17 | | 1,40 | 0,17 | | 0,68 | 0,32 | 1,31 | | 5.1x10^-08^ | | |  |  |  |  |  |  |  |
| rs57992377* | > 0.91 | 32376939 | AT | | A | 0,66 | 0,34 | | | 1,32 | 8.9x10^-08^ | 0,77 | 0,23 | 1,20 | | 0,31 | 0,81 | | 0,19 | | 1,47 | 0,12 | | 0,68 | 0,32 | 1,31 | | 4.5x10^-08^ | | |  |  |  |  |  |  |  |
| rs17718751* | > 0.99 | 32379719 | C | | T | 0,66 | 0,34 | | | 1,32 | 1.4x10^-07^ | 0,77 | 0,23 | 1,20 | | 0,30 | 0,83 | | 0,17 | | 1,41 | 0,16 | | 0,68 | 0,32 | 1,30 | | 8.7x10^-08^ | | |  |  |  |  |  |  |  |
| * imputed SNPs | |  | |  | | | |  |  | | |  | | |  | | |  | |  | | |  | | |  |  |  |  | |  |  |  |  |  |  |  |
| ^a^ Lowest quality score of imputation among all populations | | | | | | | | | | | | | | |  | | |  | |  | | |  | | |  |  |  |  | |  |  |  |  |  |  |  |
| ^b^ Effect allele | | | |  | | | |  |  | | |  | | |  | | |  | |  | | |  | | |  |  |  |  | |  |  |  |  |  |  |  |
|  |  |  | |  | | | |  |  | | |  | | |  | | |  | |  | | |  | | |  |  |  |  | |  |  |  |  |  |  |  |

| **Supplementary Table 5: Effect allele frequency of associated SNPs at loci 2q35 and 8p12 in the studied populations and in the 1000Genomes database.** | | | | | | | | | | | | |  |  |
| --- | --- | --- | --- | --- | --- | --- | --- | --- | --- | --- | --- | --- | --- | --- |
|  |  |  |  |  | | **Effect allele frequency** | | | | **Effect allele frequency in** | | | **Effect allele frequency in** | |
|  |  |  |  |  |  | **in this study** | | | | **published GWAS** ^a^ | | | **1000Genomes** | |
|  |  |  |  |  | | **Eur** | **Pol** | **Mel** | **(Pol + Mel)** | **GWAS studies** | **European** | **Asian** | **European** | **East Asian** |
|  |  |  |  |  |  |  |  |  |  |  |  |  |  |  |
|  |  |  |  | **Allele** | |  |  |  |  |  |  |  |  |  |
| **Locus** | **SNPs** | **Position** | **R info** | **Ref** | **Effect** |  |  |  |  |  |  |  |  |  |
| **2q35** | rs1169380* | 218292158 | >0.98 | G | C | 0,3 | 0,83 | 0,92 | 0,87 | Gudmundsson, 2017, Icelanders | 0,32 | - | 0,28 | 0,61 |
|  |  |  |  |  |  |  |  |  |  |  |  |  |  |  |
|  | rs1382435 | 218296003 |  | C | T | 0,35 | 0,88 | 0,98 | 0,92 | Kohler, 2013, Italian | - | - | 0,35 | 0,69 |
|  | rs6759952 | 218271719 |  | C | T | 0,44 | 0,9 | 0,98 | 0,93 |  | 0,42 | - | 0,44 | 0,77 |
|  | rs966423 | 218310340 |  | T | C | 0,43 | 0,89 | 0,98 | 0,93 | Gudmundsson, 2012, Icelanders Gudmundsson, 2017, Icelanders Son, 2017, Korean | 0,41 | - | 0,42 | 0,78 |
|  |  |  |  |  |  |  |  |  |  |  |  |  |  |  |
|  | rs1549738* | 218118722 | >0.98 | G | A | 0,14 | 0,86 | 0,94 | 0,89 | Son, 2017, Korean | - | 0,65 | 0,13 | 0,61 |
|  | rs12990503 | 218294217 |  | C | G | 0,29 | 0,83 | 0,92 | 0,88 |  | - | 0,68 | 0,28 | 0,62 |
| **8p12** | rs2466076 | 32432796 |  | T | G | 0,48 | 0,31 | 0,4 | 0,34 | Gudmundsson 2017, Icelanders | 0,48 | - | 0,48 | 0,18 |
|  | rs2439302 | 32432369 |  | C | G | 0,47 | 0,3 | 0,4 | 0,34 | Gudmundsson, 2012, Icelanders Gudmundsson, 2017, Icelanders Son, 2017, Korean | 0,48 | 0,21 | 0,48 | 0,19 |
|  |  |  |  |  |  |  |  |  |  |  |  |  |  |  |
|  | rs6996585 | 32400803 |  | A | G | 0,44 | 0,54 | 0,63 | 0,58 | Son, 2017, Korean | - | 0,23 | 0,42 | 0,24 |
|  | rs12542743 | 32318355 |  | T | C | 0,45 | 0,63 | 0,65 | 0,64 |  | - | 0,25 | 0,56 | 0,27 |
| ^a^ Effect allele frequency reported in the different published GWAS for Europeans and Asians, in controls. | | | | | | | | | | | | | |  |
| * imputed SNP | |  |  |  |  |  |  |  |  |  |  |  |  |  |
